# Transmembrane conformation of the envelope protein of an alpha coronavirus, NL63

**DOI:** 10.1002/pro.4923

**Published:** 2024-03-19

**Authors:** Iva Sučec, Yanina Pankratova, Mriganka Parasar, Mei Hong

**Affiliations:** ^1^ Department of Chemistry Massachusetts Institute of Technology Cambridge Massachusetts USA

**Keywords:** coronavirus, ion channels, magic‐angle spinning, solid‐state NMR, viroporin, water accessibility

## Abstract

The envelope (E) proteins of coronaviruses (CoVs) form cation‐conducting channels that are associated with the pathogenicity of these viruses. To date, high‐resolution structural information about these viroporins is limited to the SARS‐CoV E protein. To broaden our structural knowledge of other members of this family of viroporins, we now investigate the conformation of the E protein of the human coronavirus (hCoV), NL63. Using two‐ and three‐dimensional magic‐angle‐spinning NMR, we have measured ^13^C and ^15^N chemical shifts of the transmembrane domain of E (ETM), which yielded backbone (ϕ, ψ) torsion angles. We further measured the water accessibility of NL63 ETM at neutral pH versus acidic pH in the presence of Ca^2+^ ions. These data show that NL63 ETM adopts a regular α‐helical conformation that is unaffected by pH and the N‐terminal ectodomain. Interestingly, the water accessibility of NL63 ETM increases only modestly at acidic pH in the presence of Ca^2+^ compared to neutral pH, in contrast to SARS ETM, which becomes much more hydrated at acidic pH. This difference suggests a structural basis for the weaker channel conductance of α‐CoV compared to β‐CoV E proteins. The weaker E channel activity may in turn contribute to the reduced virulence of hCoV‐NL63 compared to SARS‐CoV viruses.

## 1 INTRODUCTION

Coronaviruses (CoVs) have caused multiple global pandemics and epidemics since 2002. These large enveloped RNA viruses infect mammals and birds, causing respiratory infections, diarrhea, and hepatitis (Fehr & Perlman, 2015; Cui et al., 2019). Because of the zoonotic potential of these viruses, it is imperative to study fundamental aspects of CoVs as a class (V'Kovski et al., 2021). While SARS and MERS CoVs have caused the worst pandemics and epidemics in the last two decades, four human CoVs (hCoVs) have been endemic since the 1960s. These include 229E, OC43, HKU1, and NL63, which together account for 15–30% of the seasonal cold infections (Harrison et al., 2023; Su et al., 2016). 229E and NL63 belong to the group of α‐CoVs, whereas OC43 and HKU1 belong to the group of β‐CoV, which also includes SARS and MERS. A third group, γ‐CoV, is represented by the avian infectious bronchitis virus (IBV), the first discovered CoV (V'Kovski et al., 2021).

Common to all CoVs are three structural membrane proteins: spike (S), membrane (M), and envelope (E) proteins (Wong & Saier Jr., 2021). The S protein adopts a petal shape on the virion surface that distinguishes CoVs from other enveloped viruses. It is responsible for binding host cell receptors to mediate CoV entry into the cell. The M protein is responsible for virus assembly in the endoplasmic reticulum Golgi intermediate compartment (ERGIC). E, the smallest of the three membrane proteins, mediates virus assembly and budding together with M and conducts cations across the ERGIC membrane (DeDiego et al., 2014). As such, E belongs to the family of viroporins, small hydrophobic membrane proteins that form ion‐conducting pores to disrupt cellular functions and cause pathogenicity (Fischer & Sansom, 2002; Nieva et al., 2012).

Since the SARS‐CoV‐1 epidemic in 2002 and the SARS‐CoV‐2 pandemic in 2019, the E proteins of these two viruses have become the best studied members of the CoV E viroporins. These two E proteins have high sequence homology and identical transmembrane (TM) sequences. The protein spans the lipid membrane once, with the N‐terminal ectodomain facing the ERGIC lumen and the C‐terminal domain in the cytoplasm (Pervushin et al., 2009; Surya et al., 2018). In detergent micelles (Li et al., 2014) and phospholipid bilayers (Somberg et al., 2022), SARS E forms pentameric helical bundles, which cluster in the presence of phosphatidylinositol and cholesterol. Functionally, the SARS E protein conducts Na^+^, K^+^, Ca^2+^, and Mg^2+^ across planar lipid bilayers, and the open probability of the channel is pH‐dependent in KCl solutions (Torres et al., 2007; Wilson et al., 2004; Wilson et al., 2006; Xia et al., 2021). Among these cations, Ca^2+^ is the only ion that has a large concentration gradient between the ERGIC lumen and the cytoplasm: the Ca^2+^ concentration is 1000‐fold higher in the ER and Golgi than in the cytoplasm (Pedriali et al., 2017). The Ca^2+^‐conducting activity of E has been linked to the inflammatory response of the host cell to SARS‐CoV infection (Murakami et al., 2012; Nieto‐Torres et al., 2014; Nieto‐Torres et al., 2015). In addition, the ERGIC lumen is acidic, whereas the cytoplasm is neutral (Appenzeller‐Herzog & Hauri, 2006).

High‐resolution structures of the TM domain of the SARS E protein were recently determined using solid‐state NMR (Mandala et al., 2020; Medeiros‐Silva et al., 2023). At neutral pH, the five‐helix bundle surrounds a narrow pore with low water accessibility, indicating that the channel is closed (Mandala et al., 2020). At acidic pH in the presence of Ca^2+^, the protein is much more water accessible, indicating that the pore is open (Medeiros‐Silva et al., 2023). In this low‐pH state, the N‐ and C‐terminal segments of SARS ETM exhibit two hydrogen‐bonded networks. The N‐terminal network features a Glu8 residue that interacts with a Thr9 residue in the neighboring helix, suggesting that these residues may be involved in proton and calcium binding. The C‐terminal network showcases an Arg38 residue whose sidechain juts into the pore, interacting with residue Thr35 in the neighboring helix. In the middle of the TM domain, three regularly spaced phenylalanine (Phe) residues form an aromatic belt on the periphery of the pentamer. The conformations of the Phe sidechains differ between the closed and open states, suggesting that the ring movement from the channel pore to the lipid interface might regulate channel gating (Medeiros‐Silva et al., 2022). Following the TM domain, the cytoplasmic domain of E shows α‐helical character when the protein is present at low concentrations in detergent micelles (Surya et al., 2018), but converts to β‐strand conformations when the protein is present in lipid bilayers at high concentrations (Dregni et al., 2023; Surya & Torres, 2022).

**FIGURE 1:**
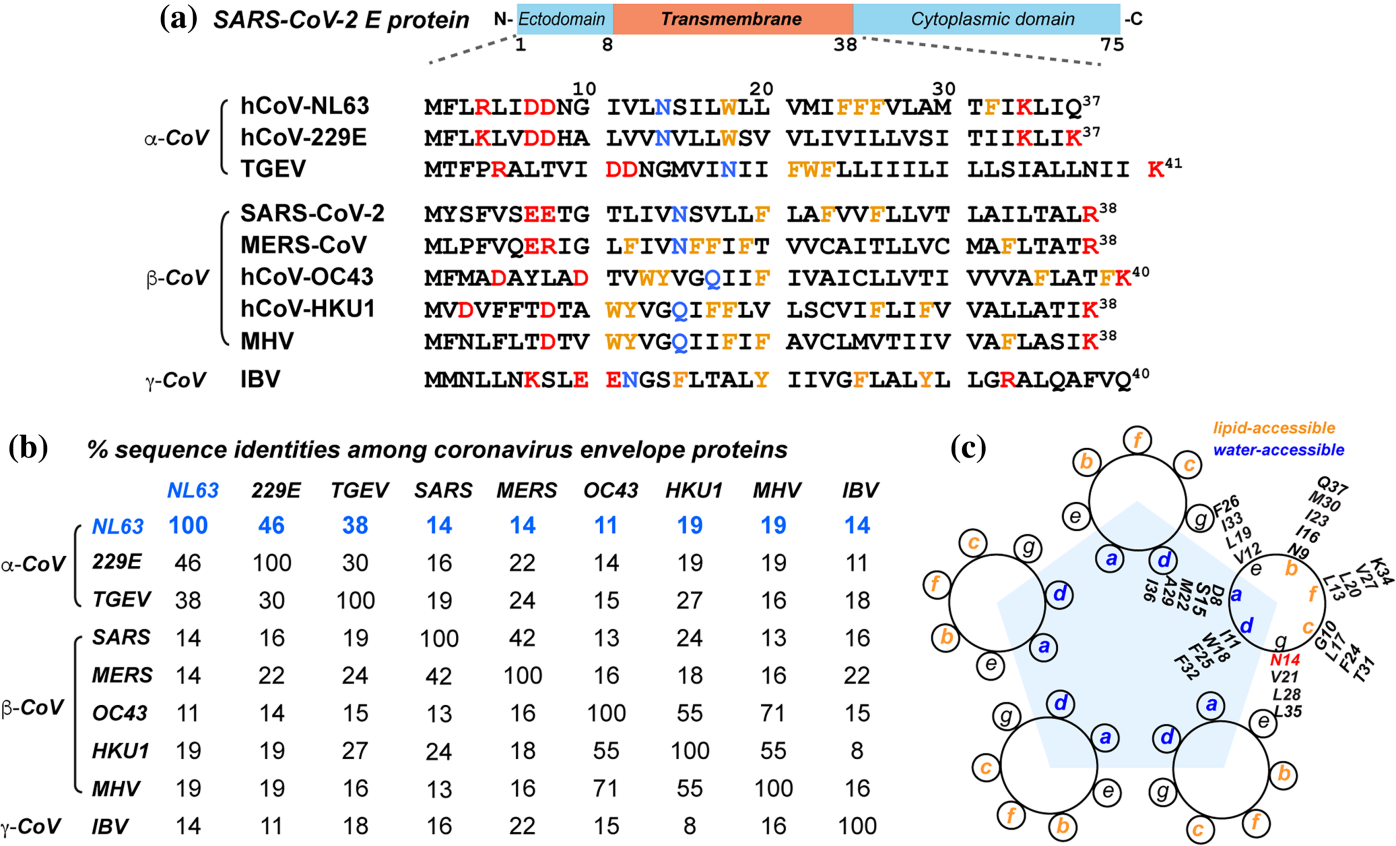
Amino acid sequence of the NL63 E protein compared to other coronaviruses' E proteins. (a) Sequence diagram of CoV E proteins and amino acid sequences of the ectodomain and TM domain of selected α‐, β‐, and γ‐CoVs. Charged residues are colored in red, aromatic residues in orange, and a conserved Asn or Gln residue in blue. (b) Percent sequence identities of the CoV E proteins. Different genera of CoVs show low sequence identities. The UniProt entry numbers for the E sequences are: NL63 (Q6Q1S0), 229E (P19741), TGEV (P09048), SARS‐CoV‐2 (P0DTC4), MERS (K9N5R3), OC43 (Q04854), HKU1 (Q0ZME5), MHV (P0C2R0), and IBV (P05139). (c) Helical wheel diagram of NL63 ETM (8–37), assuming a pentameric helical bundle with 3.5 residues per turn. Residues at heptad positions *a* and *d* are fully water accessible, whereas residues at heptad positions *b*, *c*, and *f* are lipid‐accessible. Positions *e* and *g* are interfacial and partly water accessible. Residue N14 is at interfacial heptad position *g*, based on previous water‐ and lipid‐edited ssNMR data (Sučec et al., 2023).

In contrast to SARS, no high‐resolution structural information about the E proteins of other CoVs is so far available. All E proteins have a short and hydrophilic N‐terminus, followed by the TM domain and a C‐terminal cytoplasmic tail (Figure 1a). Two to four cysteines are found in the membrane‐proximal region of the cytoplasmic domain. Despite these common amino acid sequence motifs, the functions of the E proteins differ among different CoVs (González et al., 2003). For example, E knockout from the mouse hepatitis virus (MHV), a β‐CoV, can be rescued by E proteins of other β‐ and γ‐CoVs but not by the E protein of the α‐CoV transmissible gastroenteritis virus (TGEV) (Kuo et al., 2007). Different CoVs' E proteins also differ in ion selectivity. For example, SARS, MHV, and IBV E proteins form ion channels with higher selectivity to Na^+^ than to K^+^, whereas the E protein of 229E, an α‐CoV, is more selective for K^+^ than Na^+^ (Wilson et al., 2004; Wilson et al., 2006). These distinct biochemical properties suggest that the structures of E viroporins have detailed differences among different CoVs.

One of the globally circulating α‐CoVs is NL63. This virus was first isolated from an infant in the Netherlands with acute respiratory illness soon after the first SARS epidemic (van der Hoek et al., 2004). Phylogenetic analysis indicates that NL63 has long been circulating in human populations, diverging from its closest relative 229E in the eleventh century (Pyrc et al., 2006). The virus causes mild upper respiratory symptoms such as cough and fever as well as more severe lower respiratory symptoms such as bronchiolitis and croup (Abdul‐Rasool & Fielding, 2010). The N‐terminal ectodomain and TM domain of NL63 E have sequence identities of 38–46% with E proteins of the α‐CoVs TGEV and 229E, but less than 20% sequence identity with E proteins of β‐ and γ‐CoVs such as SARS and MERS (Figure 1b). While SARS, MERS, and IBV E proteins have one or two Glu residues at the N‐terminal region of the TM domain, NL63 and 229E E proteins have two Asp residues at the corresponding positions. At the C‐terminal end of the TM domain, SARS, MERS, and IBV E proteins have an Arg residue, whereas NL63 and 229E E proteins have a Lys residue. NL63 E has three consecutive Phe residues in the middle of the TM domain, whereas β‐CoV E proteins intersperse their aromatic residues with aliphatic residues. Because the SARS ETM structures implicate charged residues to be crucial for calcium and pH sensitivity and aromatic residues to be important for channel gating, these amino acid sequence differences suggest that NL63 E may have detailed structural differences from the SARS E protein.

To understand the structure and function of E proteins from different CoV groups, here we investigate the structure of the membrane‐bound NL63 E protein. Using ^13^C and ^15^N magic‐angle‐spinning (MAS) NMR, we have measured the conformation, helical packing, and water accessibility of NL63 ETM in lipid bilayers. ^13^C and ^15^N chemical shifts obtained from 2D and 3D correlation NMR spectra define the α‐helical TM portion of the protein. Comparison of the water accessibilities of NL63 ETM at high and low pH reveals interesting differences from the SARS‐CoV‐2 ETM, suggesting a structural basis for the functional differences between α‐ and β‐CoV E proteins.

## 2 RESULTS

### 2.1 NL63 ETM is α‐helical and the conformation is unaffected by the ectodomain

Here, we report the first characterization of the conformation of the E protein of an α‐CoV, NL63. The study is motivated by the fact that E viroporins of different CoVs differ significantly in the positions of polar, charged, and aromatic residues in the amino acid sequences (Figure 1). Thus, the structure of the E protein of SARS‐CoV‐2, a β‐CoV, may not accurately represent the E proteins of other groups of CoVs, and direct structural studies are needed.

**FIGURE 2:**
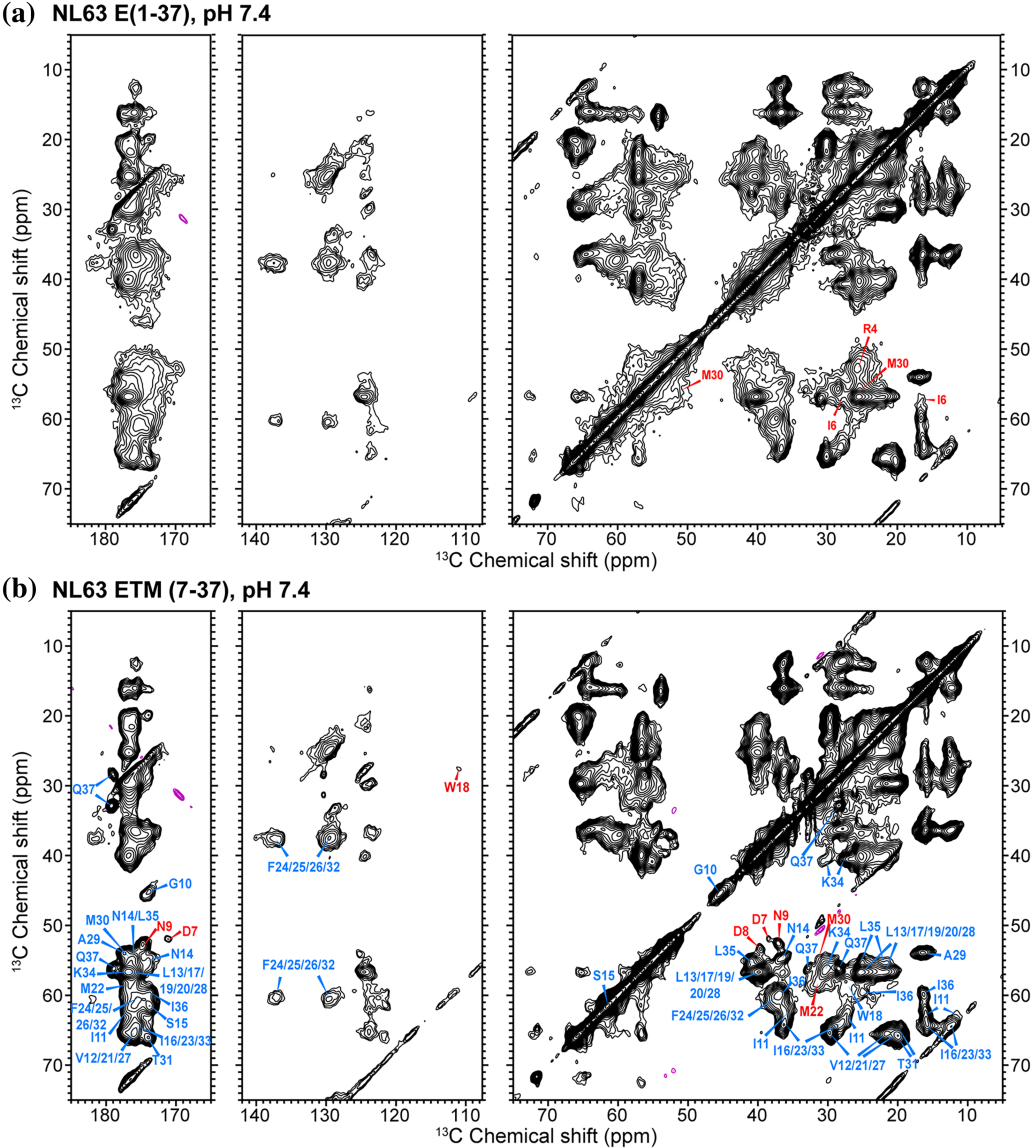
2D ^13^C‐^13^C correlation spectra of membrane‐bound NL63 E proteins at pH 7.4. (a) E (1–37) spectrum. (b) ETM (7–37) spectrum. Both spectra were measured under 10.5 kHz MAS using a CORD mixing time of 25 ms. Resonance assignments are obtained from 3D correlation spectra. Spectra were processed using Gaussian line broadening with parameters lb/gb = −10/0.03 for both dimensions. Assignments transferrable between the two spectra are shown in blue in (b), while assignments that are different or available only for one of the two proteins are shown in red. The protein was reconstituted in the POPX/cholesterol membrane. 2D ^13^C‐^13^C correlation spectrum of NL63 E(1–37) was acquired at 22°C while spectrum of ETM was at 25°C.

**FIGURE 3:**
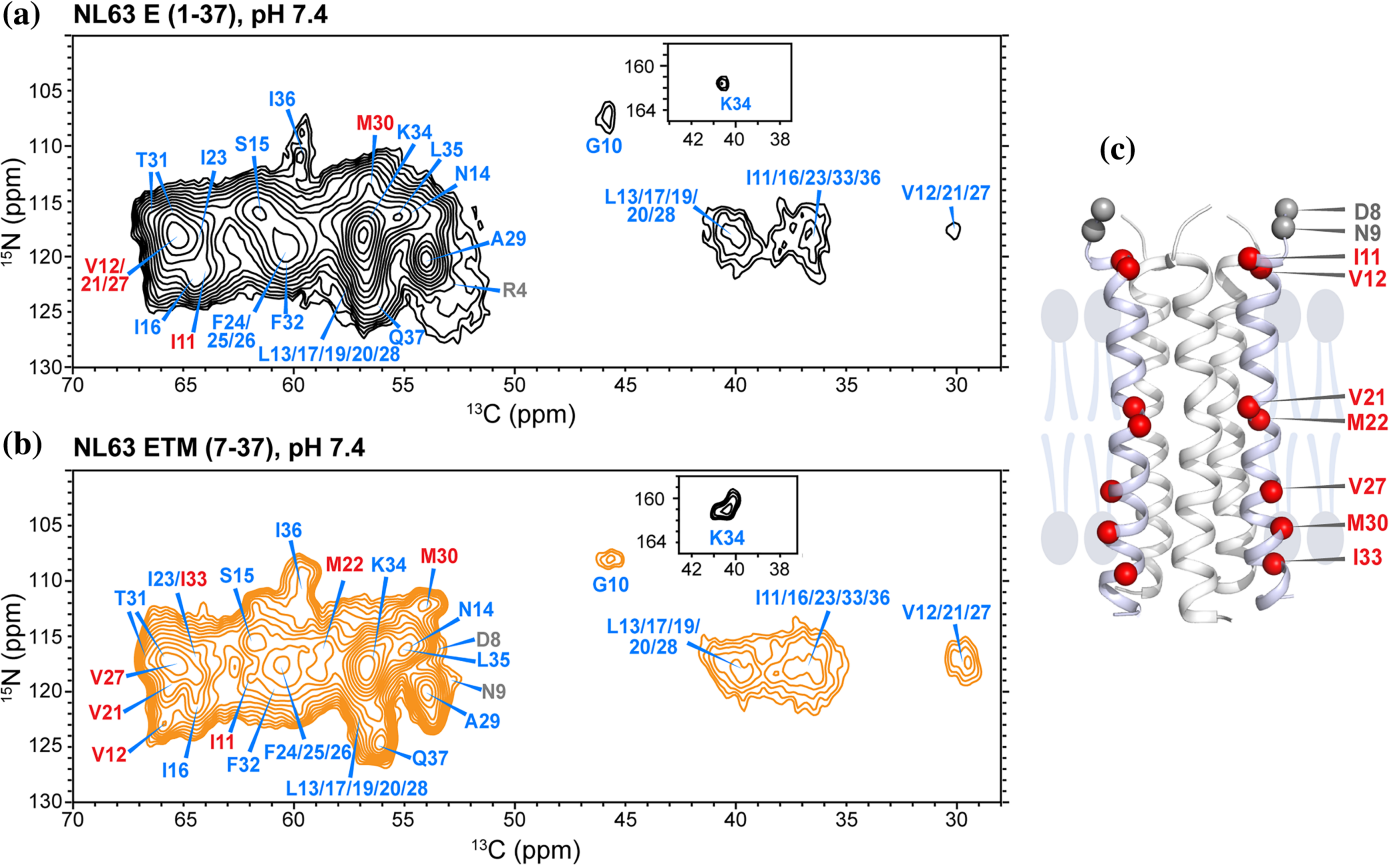
2D ^15^N‐^13^C correlation spectra of membrane‐bound NL63 E proteins at pH 7.4, measured between 22°C and 25°C. (a) E (1–37) spectrum. (b) ETM spectrum. Resonance assignments are obtained from 3D correlation experiments. The spectra were processed using a qsine function with ssb = 2.5 for both dimensions. Assignments transferrable between the two spectra are shown in blue, assignments that differ between the two samples are shown in red, and assignments that are available only for one of the two proteins are shown in gray. Insets show the folded resonances of the K34 sidechain. (c) Topology model of NL63 ETM obtained from water‐ and lipid‐edited methyl NMR data (Sučec et al., 2023). Residues with different assignments between E(1–37) and ETM are shown as red spheres while residues for which assignment is available only for one of the two samples are shown in gray.

We investigated the conformation of two NL63 E constructs: ETM (7–37) and E (1–37). The latter contains the N‐terminal ectodomain in addition to the TM domain. Both proteins were reconstituted into a POPX/cholesterol membrane, which differs from our previous ERGIC‐mimetic membrane by the removal of phosphatidylinositol to avoid protein clustering (Somberg et al., 2022). Both constructs were studied at pH 7.4; in addition, ETM was also studied at pH 4.5 in the presence of Ca^2+^. For simplicity, we refer to the latter condition as the low‐pH condition. 2D ^13^C‐^13^C and ^15^N‐^13^C correlation spectra (Figures 2 and 3) of the long and short constructs at pH 7.4 show ^13^C linewidths of 1.1–1.7 ppm and ^15^N linewidths of 2–3 ppm. These linewidths are much broader than those of SARS‐CoV‐2 ETM (Mandala et al., 2020; Medeiros‐Silva et al., 2022), indicating that NL63 E is conformationally more disordered than SARS E. The NL63 E spectral resolution is more comparable to that of the influenza A M2 protein (AM2), which is known to be conformationally plastic (Cady et al., 2009; Kwon et al., 2015; Kwon & Hong, 2016). Removal of the ectodomain moderately improved the spectral resolution, resolving residues such as D7, D8, and N14 in the 2D CC spectrum (Figure 2b), which otherwise overlap with the signals of the ectodomain. However, the rest of the ETM signals remain poorly resolved in the 2D spectra.

**FIGURE 4:**
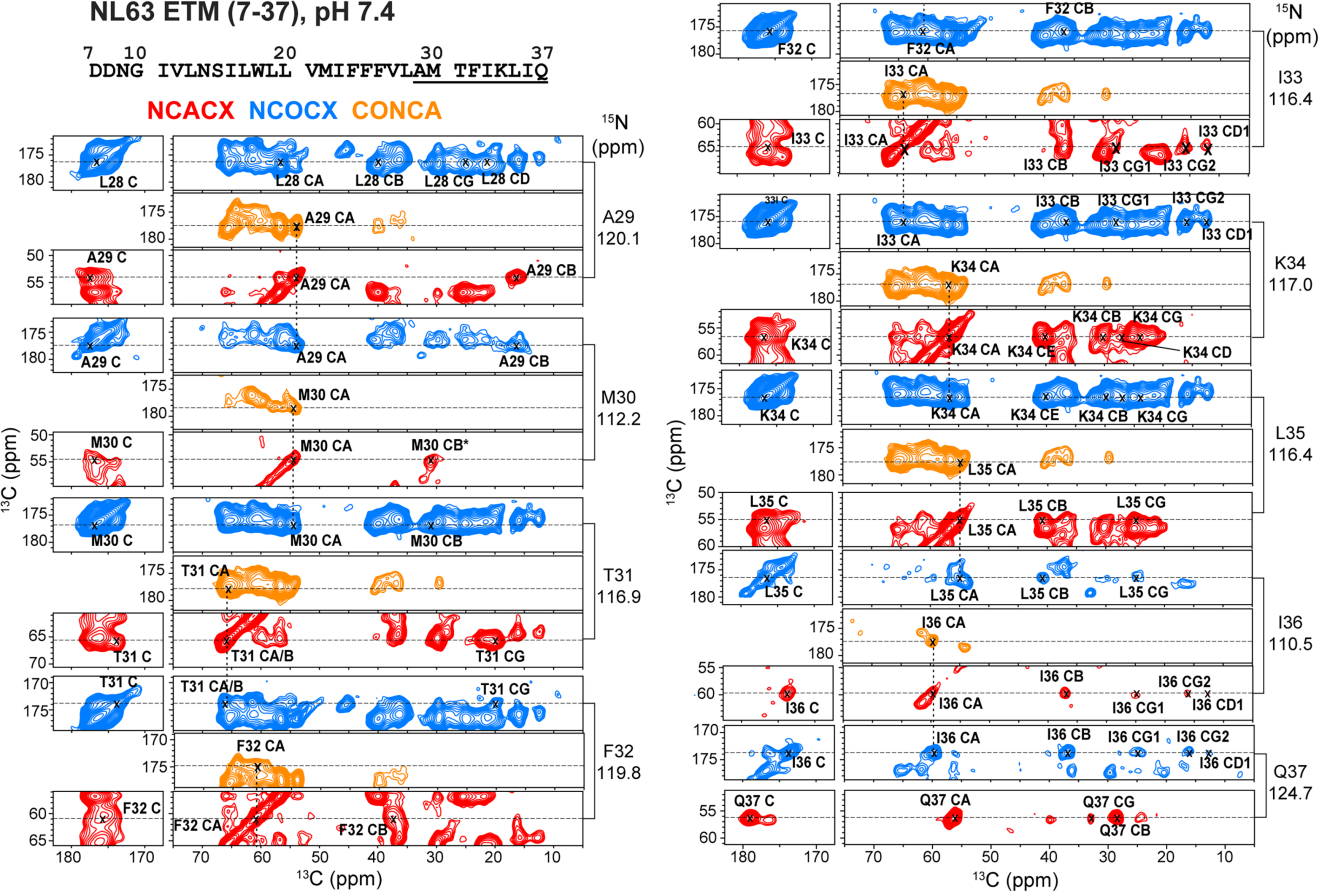
Representative strips of 3D NCACX, NCOCX, and CONCA spectra of NL63 ETM at pH 7.4. Spectra were processed using Gaussian line broadening with parameters lb/gb = −20/0.03 for the direct dimension and lb/gb = −30/0.02 for the two indirect dimensions. The strips of residues A29 to Q37 are shown (underlined in the sequence). The M30 Cβ chemical shift (marked with an asterisk) is indicative of a reduced sidechain.

To assign the chemical shifts of E (1–37) and ETM (7–37) at pH 7.4, we utilized 3D NCACX, NCOCX, and CONCA spectra (Figures 4 and S1a) in addition to the 2D CC and NC spectra. The 3D NCACX spectrum of ETM displays the spin systems of most residues, indicating that the protein is immobilized in the POPX/cholesterol membrane. Several features of the spectra facilitated the assignment of ETM. First, the 2D CC spectrum of ETM resolved the ^7^DDNG^10^ signals, which are obscured in the E (1–37) spectrum by the R4 signals (Figure 2b). Second, a cross peak between the G10 ^15^N chemical shift and the N9 ^13^CO chemical shift was observed in the NCOCX spectrum of ETM but not E (1–37). This suggests that these two residues are immobilized in ETM but are dynamically disordered in E (1–37). Finally, ETM shows moderately improved ^15^N chemical shift resolution compared to E (1–37), which allowed the assignment of some of the Val and Ile signals. For both E (1–37) and ETM, the ^15^N chemical shifts of I36 and Q37 are well resolved from the other ^15^N peaks, thus their 3D strips do not overlap with the other residues. In total, we assigned all 31 residues in ETM and 30 out of 37 residues in E (1–37) (Tables S1 and S2).

The assigned ^13^C and ^15^N chemical shifts indicate that the E ectodomain has little impact on the TM conformation: the chemical shift perturbations (CSPs) between the two constructs are <0.5 ppm for most TM residues (Figure S1b, Table S3). The exceptions are D7, I11, V12, and M30. We attribute the larger CSPs of residues N7 to V12 to their interactions with the ectodomain and the M30 CSP to sidechain oxidation in E (1–37). Oxidized Met has typical Cβ and Cγ chemical shifts of about 52 ppm and 25 ppm (Hinterholzer et al., 2020), whereas reduced Met has overlapping Cβ and Cγ chemical shifts of about 35 ppm. We observed split Cβ and Cγ chemical shifts for M30 in the E (1–37) sample (Figure S1a), indicative of M30 oxidation. However, this oxidation did not affect the conformation of the neighboring A29 and T31, as no significant CSPs were observed (Figure S1b). Finally, a 1D ^13^C INEPT spectrum of NL63 E (1–37) (Figure S2) reveals protein signals that can be attributed to residues of the ectodomain and/or the C‐terminal three residues, such as Met (Cγ at ~51 ppm), Leu (Cα at 56.8 and 56.2 ppm, Cβ at 40.5 ppm and Cδ at 19.6 ppm), and Ile (Cα at 62.2 ppm), indicating that the ectodomain is dynamically disordered.

**FIGURE 5:**
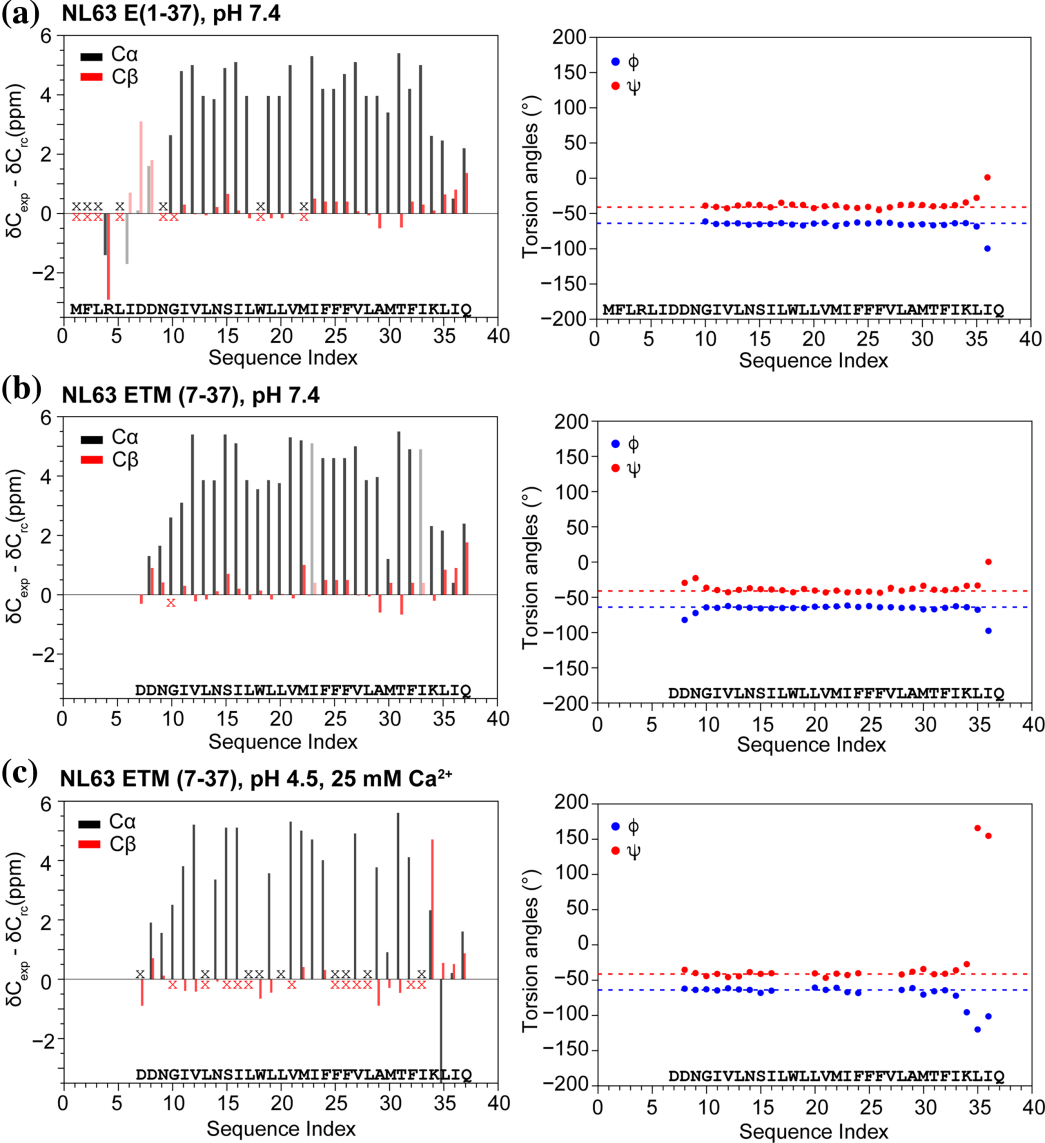
Cα and Cβ secondary chemical shifts (left column) and resulting TALOS (ϕ, ψ) angles (right column) of membrane‐bound NL63 E. (a) E (1–37) at pH 7.4. (b) ETM (7–37) at pH 7.4. (c) ETM (7–37) at pH 4.5 with 25 mM Ca^2+^. Shaded bars in the left column denote ambiguous chemical shifts. Black and red “x” symbols indicate unassigned chemical shifts. The similarities of the chemical shifts and torsion angles indicate that the ectodomain has minimal impact on the TM conformation of the protein. Dashed lines guide the eye for the approximate (ϕ, ψ) angles of ideal α‐helices.

For residues from G10 to K34, the measured Cα chemical shifts are larger than the random coil values, whereas the Cβ chemical shifts are smaller (Figure 5), indicating that this segment is α‐helical. TALOS‐N (Shen & Bax, 2015) prediction of backbone (ϕ, ψ) angles based on the measured ^13^C and ^15^N chemical shifts (Tables S1 and S2) confirms that this domain adopts a regular α‐helical conformation. Residues ^7^DDN^9^ in ETM but not E (1–37) show small deviations from ideal helical torsion angles, indicating that the ectodomain stabilizes the helical conformation of these residues. In both constructs, the helical propensity decreased after K34, suggesting that these residues (^34^KLIQ^37^) may belong to the cytoplasmic domain rather than the TM domain.

### 2.2 Helix packing topology of NL63 ETM

**FIGURE 6:**
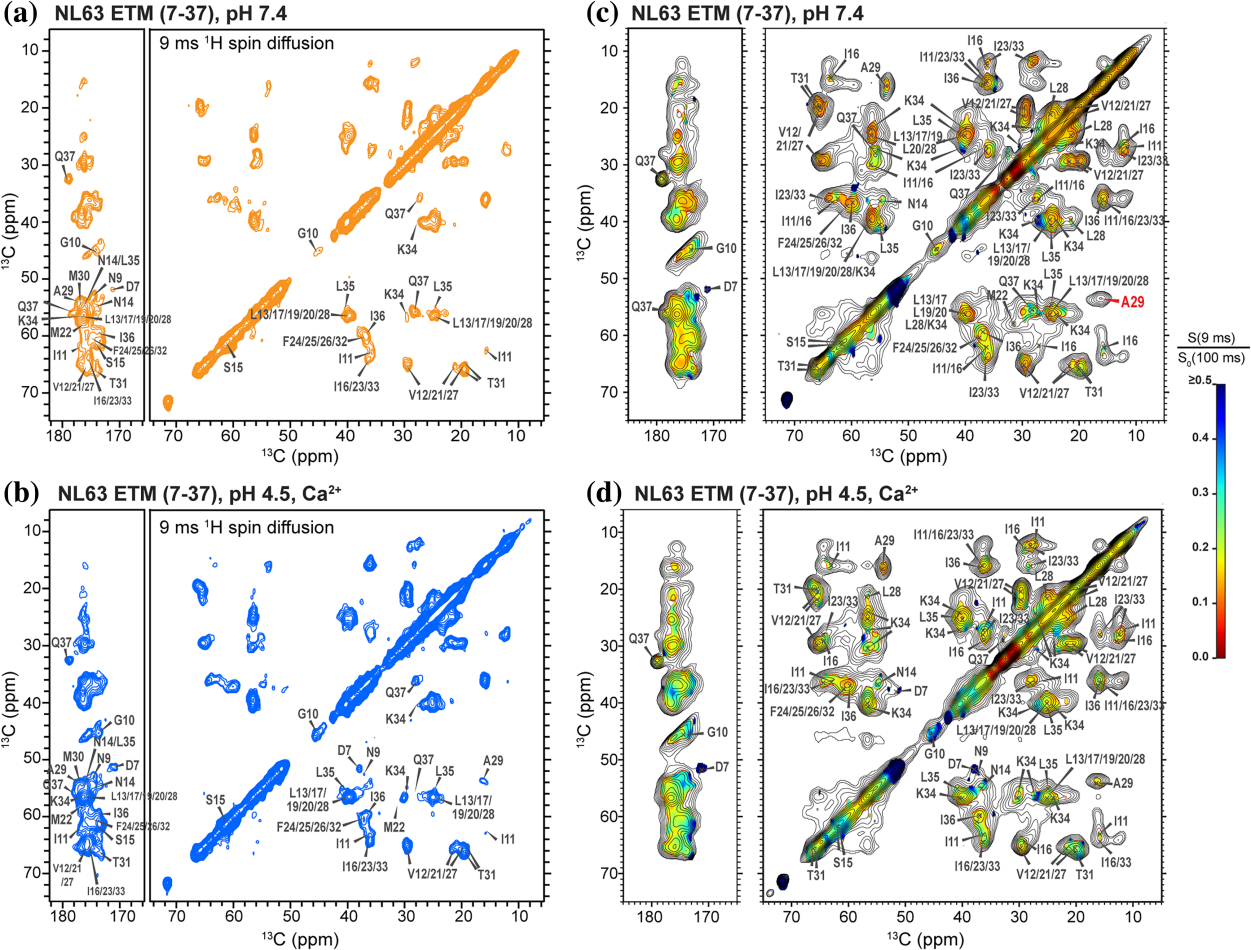
Water accessibilities of NL63 ETM at high and low pH from water‐edited 2D CC spectra, measured at 25°C. (a) 9 ms water‐edited 2D CC spectrum of membrane‐bound NL63 ETM at pH 7.4. (b) 9 ms water‐edited 2D CC spectrum of the pH 4.5 sample with Ca^2+^. (c) Hydration plot of the pH 7.4 sample. (d) Hydration plot of the pH 4.5 Ca^2+^ − bound sample. Color bar represents the ratios of 9 ms and 100 ms water‐transferred intensities. Gray contours show the unedited 2D CC spectrum. The low‐pH protein exhibits moderately higher water accessibilities than the high‐pH protein. Assignments of signals used for extracting residue‐specific water accessibility values are indicated.

To investigate the helix‐packing topology of NL63 ETM and probe whether this topology is affected by the ectodomain, we measured the water accessibilities of ETM at pH 7.4 using a water‐edited 2D CC experiment (Figure 6a,c). Residue‐specific water‐transferred intensities provide information about the radial positions of the amino acid residues with respect to the channel pore. Pore‐facing residues should exhibit higher water‐transferred intensities than lipid‐facing residues. We first applied a recently developed approach that utilizes the high‐intensity Ile, Val, and Leu methyl peaks in water‐edited CC spectra (Sučec et al., 2023) to deduce the helix packing topology. Using this approach, we previously showed that NL63 E (1–37) adopts a topology in which N14 lies at the ***g*** position of the heptad repeat (N14*g*). The water‐edited 2D CC spectra of E (1–37) indicate either an N14*d* topology or an N14*g* topology, while lipid‐edited ^13^C spectra favor the N14*g* topology (Sučec et al., 2023). Here, the 9 ms water‐edited 2D CC spectrum of ETM shows similar methyl ^13^C intensity distributions as E (1–37) (Table S4), indicating that ETM adopts the same N14*g* topology as E (1–37).

**FIGURE 7:**
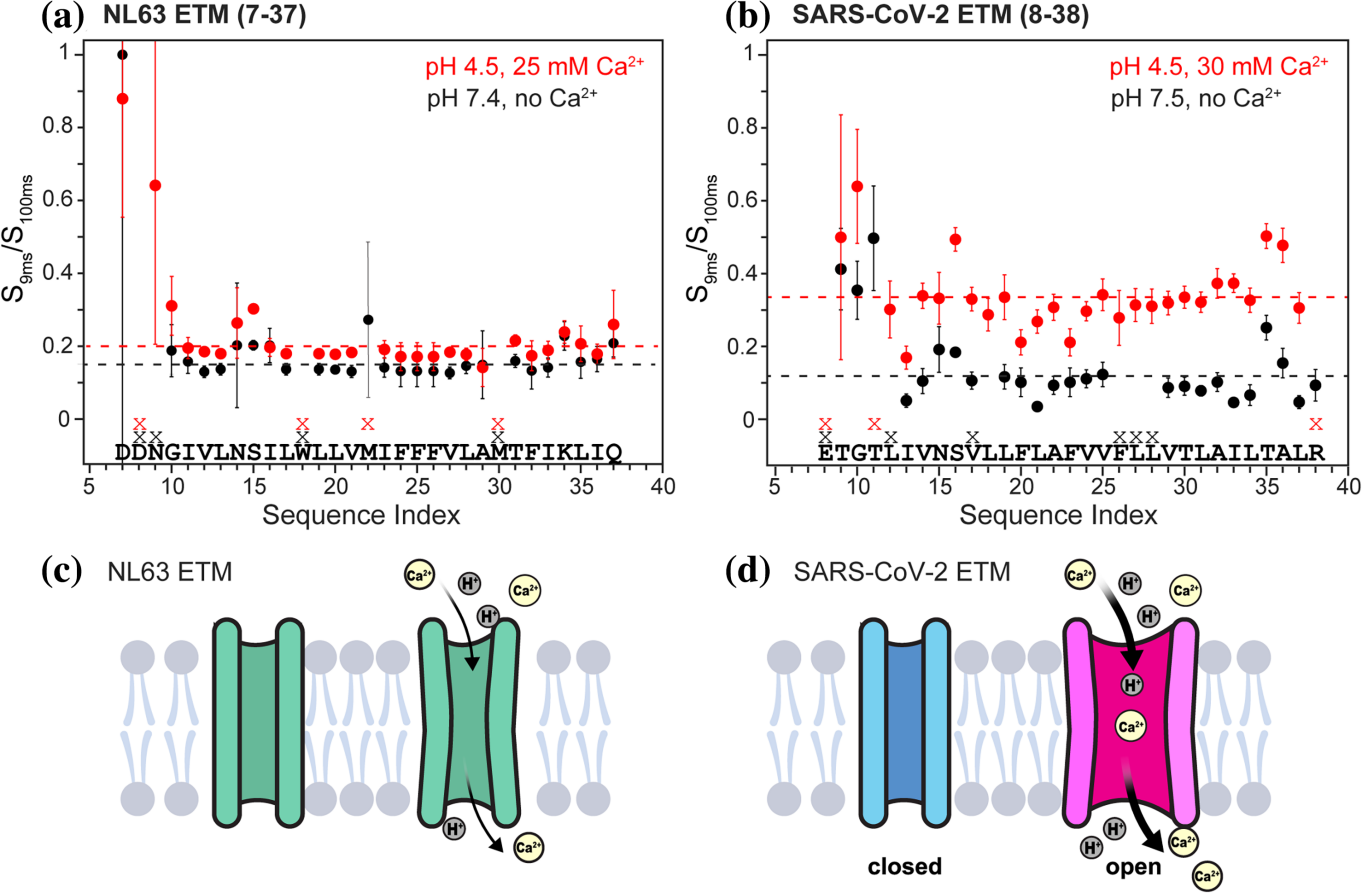
NL63 and SARS‐CoV‐2 ETM proteins have distinct water accessibility changes at low pH in the presence of Ca^2+^. The water accessibilities are obtained from water‐edited 2D CC spectra. (a) NL63 ETM water accessibilities at pH 7.4 (black) and pH 4.5 with 25 mM Ca^2+^ (red), measured at 25°C. (b) SARS‐CoV‐2 ETM water accessibilities at pH 7.5 (black) and pH 4.5 with 30 mM Ca^2+^ (red) (Medeiros‐Silva et al., 2023), measured at 13°C. NL63 ETM exhibits much smaller acid‐ and Ca^2+^‐induced water accessibility increase compared to SARS‐CoV‐2 ETM. Residues whose signals are overlapped or unassigned are indicated by the symbol “x.” Error bars were propagated from the spectral noise. (c) Schematic model of the pH‐ and Ca^2+^‐induced NL63 ETM structural change. Acidic pH and Ca^2+^ induce only modest changes in the water accessibility. (d) Schematic model of the pH‐ and Ca^2+^‐induced SARS‐CoV‐2 ETM structural change (Medeiros‐Silva et al., 2023).

More detailed information about the water accessibilities of individual residues can be obtained by analyzing the intensity ratios of resolved peaks in the water‐edited 2D CC (Figure 7a) and 2D NC spectra (Figure S3) between 9 ms and 100 ms ^1^H spin diffusion. While the water‐edited 2D CC experiment detects the hydration of both the protein backbone and sidechains, the water‐edited 2D NC experiment detects the hydration of the protein backbone after magnetization transfer from water to amide protons. At high pH, both experiments show low intensity ratios of ~0.15 for the majority of the TM segment from I11 to K34, whereas the N‐ and C‐terminal residues exhibit higher water accessibilities (Tables S5 and S6).

### 2.3 NL63 ETM hydration does not increase significantly at acidic pH in the presence of Ca^2+^

**FIGURE 8:**
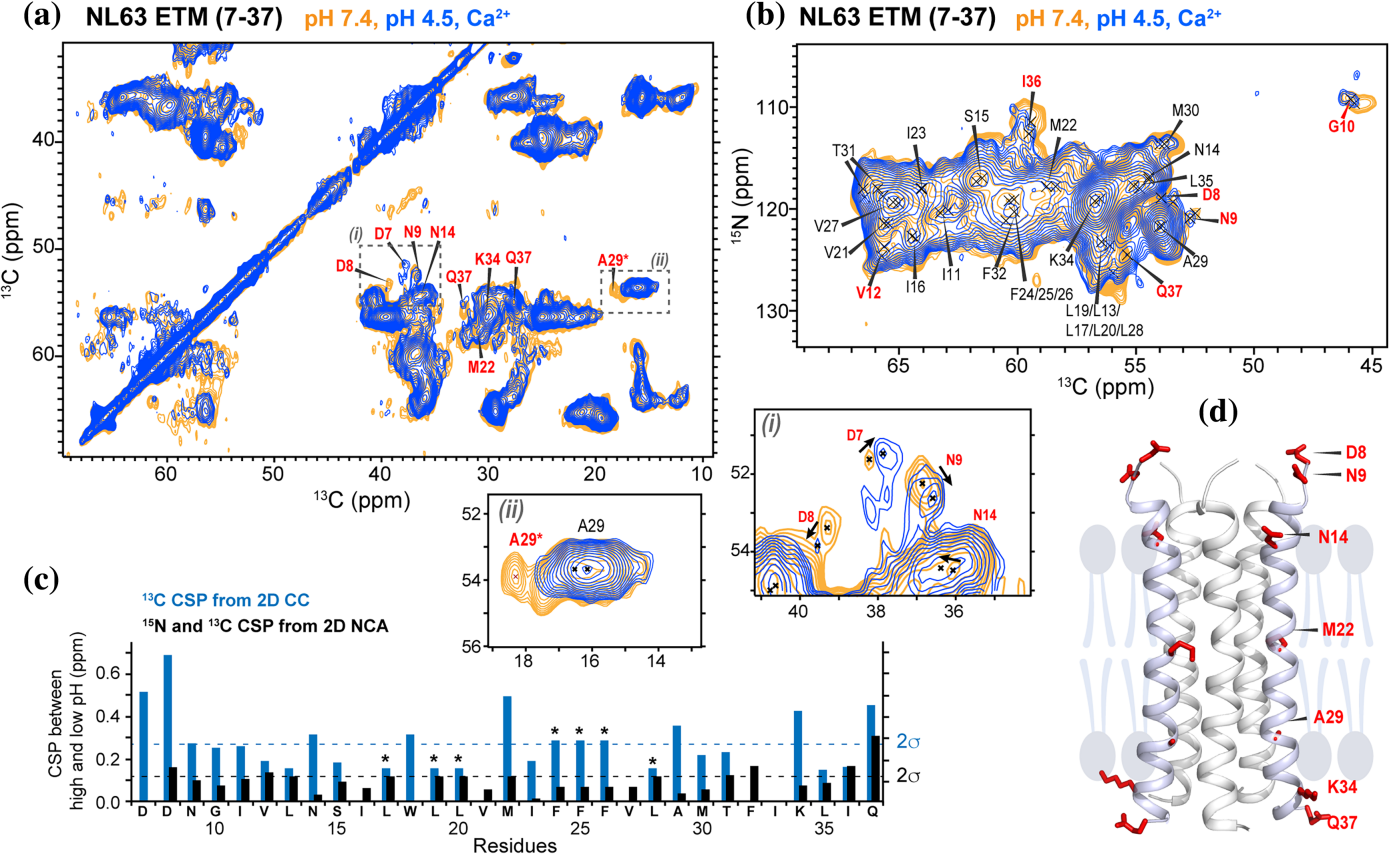
2D ^13^C‐^13^C and ^15^N‐^13^C correlation spectra of NL63 ETM (7–37) at pH 7.4 and pH 4.5 with Ca^2+^. (a) 2D CC spectra with 24 ms CORD mixing. Residues assigned in red have visible chemical shift perturbations (CSPs) between the two samples. Zoomed‐in areas show some of the CSPs more clearly. (b) 2D NC spectra. Residues assigned in red are resolved peaks that show visible chemical shift changes between the two samples. The spectra were processed with qsine line broadening with ssb = 3 applied to both dimensions. (c) ^13^C and ^15^N CSPs between the high‐pH and low‐pH NL63 ETM samples. Overlapped residues are marked with asterisks. (d) Topological model of membrane‐bound NL63 ETM. Residues with significant chemical shift perturbations between the high‐pH and low‐pH conditions are shown as red sticks.

The high‐resolution structures of SARS‐CoV‐2 ETM show that the protein widens its pore on the N‐terminal side and increases its water accessibility at low pH compared to the high‐pH state (Medeiros‐Silva et al., 2023). Since sufficient pore diameter and hydration are necessary for ion conduction, this pH‐dependent structural change is consistent with the cation‐conducting function of SARS‐CoV‐2 E. To investigate if NL63 ETM opens under similar conditions, we prepared an ETM sample at pH 4.5 with 25 mM Ca^2+^ and compared its water accessibility with the pH 7.4 sample. 2D ^13^C‐^13^C and ^15^N‐^13^C correlation spectra show that the low‐pH ETM sample has the same chemical shifts (Figure 8, Table S2) and TALOS (ϕ, ψ) angles (Figure 5b,c) as the high‐pH sample within experimental uncertainty, indicating that the backbone conformation is unchanged by pH.

Water‐edited 2D CC spectra of the low‐pH NL63 ETM (Figure 6b,d) show that the residue‐specific water‐transferred intensities increased from an average value of ~0.16 at high pH to ~0.21 at low pH (Figure 7a, Table S5). The moderately increased water accessibility is not caused by helix packing differences at low pH, as the methyl ^13^C intensities in the water‐edited 2D CC spectra and lipid‐to‐protein magnetization transfer rates (Figure S4) are similar between the two pH conditions. Importantly, the extent of water accessibility increase of NL63 ETM is much smaller compared to SARS‐CoV‐2 ETM, which shows an average S/S_0_ value of ~0.12 at high pH and ~ 0.33 at low pH (Figure 7b).

The water‐edited experiments for NL63 ETM were conducted at 25°C, whereas the SARS‐CoV‐2 water‐edited 2D CC spectra were measured at 13°C. Water magnetization transfer to proteins requires chemical exchange and ^1^H spin diffusion (Doherty & Hong, 2009; Lesage et al., 2006; Lesage & Bockmann, 2003), whose rates are temperature dependent. From 13°C to 25°C, chemical exchange rates increase several fold (Liepinsh & Otting, 1996), accelerating ^1^H magnetization transfer from water to amide and hydroxyl protons in the protein. In comparison, dipolar ^1^H spin diffusion is expected to be similar for the membrane‐bound E proteins, which are similarly immobilized within this temperature range. Thus, the higher temperature for the NL63 ETM experiments should increase the water‐edited spectral intensities compared to SARS ETM. Instead, we found a smaller low‐pH‐induced increase in water‐transferred intensities for NL63 ETM than for SARS ETM, indicating that NL63 E is less responsive to low pH and calcium ions for pore opening. Taken together, these water‐edited NMR data indicate that NL63 ETM has distinct structural responses to pH and calcium from SARS‐CoV‐2 ETM.

## 3 DISCUSSION

### 3.1 The α‐helical conformation of NL63 ETM is insensitive to the ectodomain and pH

The data shown here represent the first detailed structural characterization of the E protein of a human α‐CoV, which allows comparison with the structure of the E protein of the β‐CoV SARS (Mandala et al., 2020; Medeiros‐Silva et al., 2023). Our results define the α‐helical conformation and helical bundle topology of NL63 ETM, delineate the TM boundary more precisely than predicted by sequence alignment, and reveal important differences in the pH and calcium response of NL63 ETM from SARS‐CoV‐2 ETM.

The ^13^C and ^15^N chemical shifts of the NL63 E constructs at high and low pH and with and without the ectodomain indicate that the hydrophobic segment from G10 to K34 adopts a regular α‐helical conformation that is largely unaffected by pH and the ectodomain (Figure 5). The chemical shifts do not show discernible helical disorder in the middle of the TM segment, where three consecutive Phe residues (^24^FFF^26^) are found. This regular α‐helicity differs from the conformation of SARS‐CoV‐2 ETM, which shows helical disorder in the middle of the TM domain where three regularly spaced Phe residues (^20^FxxFxxF^26^) are present (Medeiros‐Silva et al., 2022). The ectodomain of NL63 E does not affect the TM conformation, except for residues 7–12, which exhibit small chemical shift changes. In all three samples, residues C‐terminal to K34, ^35^LIQ^37^, are less α‐helical, their torsion angles and chemical shifts vary with pH, and their water accessibility is insensitive to pH changes. These results suggest that these three residues lie outside the membrane and are better categorized as part of the cytoplasmic domain. Thus, the TM domain of NL63 E is slightly shorter than predicted by sequence alignment with SARS‐CoV‐2 ETM.

The dipolar ^13^C and ^15^N spectra obtained here do not show the resonances of most of the ectodomain residues, including M1, F2, L3, and L5, indicating that the ectodomain is mobile. This mobility is further supported by the ^13^C INEPT spectrum, which shows non‐lipid peaks that can be assigned to some of the residue types in the ectodomain (Figure S2). The mobility of the NL63 E ectodomain is similar to that of the influenza A M2 ectodomain. ^13^C NMR spectra of AM2 (residues 1–49) measured using cross polarization (CP) and direct polarization (DP) showed sharp signals for the ectodomain residues 1–21 in the DP spectra and weak intensities in the CP spectra. Moreover, the ectodomain's mobility decreases toward the TM domain (Kwon & Hong, 2016). Interestingly, the AM2 ectodomain perturbed the TM chemical shifts in a way that is similar to the chemical shift perturbation caused by the M2 inhibitor, amantadine (Cady et al., 2009; Cady et al., 2010). This result indicates that the AM2 ectodomain shifts the conformational equilibrium of the TM domain toward the drug‐binding competent state. In comparison, the NL63 E ectodomain is much shorter than the AM2 ectodomain and does not exert noticeable effects on the TM conformation.

The NL63 ETM spectra shown here were measured on a mixed membrane containing POPC, POPE, POPS, and cholesterol. This membrane differs from the ERGIC‐mimetic membrane used to reconstitute SARS ETM, which included phosphatidylinositol (PI) (Mandala et al., 2020; Medeiros‐Silva et al., 2022; Medeiros‐Silva et al., 2023). Our recent measurement of the oligomeric state of SARS ETM using ^19^F spin diffusion NMR experiments found that SARS ETM pentamers are clustered in PI‐containing membranes, but the clustering is removed when PI is excluded from the membrane (Somberg et al., 2022). However, PI does not change the backbone conformation of SARS ETM, as shown by the lack of chemical shift perturbation between DMPC/DMPG‐bound protein and ERGIC‐bound protein (Medeiros‐Silva et al., 2022). These results suggest that the α‐helical conformation and channel pore hydration of NL63 ETM should be applicable to other membranes, regardless of whether the NL63 ETM pentamers cluster or not.

### 3.2 Channel conductance differences among CoV E proteins

Electrophysiological data have been reported not only for SARS‐CoV E but also for the E proteins of several other coronaviruses, including the β‐CoVs MHV and MERS, the γ‐CoV IBV, and the α‐CoV 229E (Surya et al., 2015; Wilson et al., 2006). The 229E and NL63 E proteins have a high sequence homology of 46% (Figure 1b): they contain the same double Asp's at the N‐terminal entrance of the TM domain, the same polar Asn at residue 14, the same Trp at residue 18 in the middle of the TM domain, and the same Lys at residue 34 capping the C‐terminal end of the TM domain. Thus, we use the channel activity of the 229E E protein as a proxy for the NL63 E channel function.

All these CoV E viroporins conduct cations, but their specific cation conductance, permeability, and selectivity differ, implying that these proteins have detailed differences in their membrane‐bound structures. The SARS‐CoV ETM is selective for monovalent and divalent cations over anions (Wilson et al., 2004; Xia et al., 2021), and the cation conductance is dependent on pH (Verdia‐Baguena et al., 2013; Xia et al., 2021) and membrane surface charge (Nieto‐Torres et al., 2015). Single‐channel recording shows that SARS‐CoV ETM is more permeable to Na^+^ than K^+^, and these permeabilities are several fold larger than those of divalent Ca^2+^ and Mg^2+^ (Xia et al., 2021). The K^+^ conductance of SARS ETM is sensitive to pH: the open probability increased from less than 5% at pH 8 to ~20% at pH 4, and the current amplitude increased from ~2 pA to ~5 pA over the same pH interval (Xia et al., 2021). In comparison, the E proteins of MHV and IBV show similar ion permeability profiles as SARS at pH 7.2, favoring Na^+^ over K^+^, whereas the 229E E protein is more permeable to K^+^ than Na^+^ (Wilson et al., 2006). The K^+^ conductance of 229E E (23 ± 5 pS) is several fold smaller than that of SARS‐CoV ETM (93 ± 36 pS) (Wilson et al., 2004). Since the 229E E protein is less permeable to Na^+^ than K^+^ while the SARS‐CoV ETM is more permeable to Na^+^ than K^+^, the 229E viroporin is overall less efficient in ion conduction than the SARS viroporin.

These channel activity differences correlate well with the NMR‐detected pH‐ and Ca2^+^ ‐dependent water accessibilities of NL63 and SARS ETM proteins. The water‐edited ssNMR spectra showed that SARS‐CoV‐2 ETM is much more water accessible at pH 4.5 in the presence of Ca^2+^ than at pH 7.5 (Figure 7b). At the same time, distance‐ and orientation‐restrained high‐resolution structures of SARS‐CoV‐2 ETM show that the low‐pH protein has a large water‐filled chamber for the N‐terminal half of the TM domain that is capped with Glu8 and a Thr9 (Medeiros‐Silva et al., 2023). This water‐filled helical barrel is distinct from the high‐pH structure of the protein, which is a tight five‐helix bundle for most of the TM domain (Mandala et al., 2020). In contrast, NL63 ETM shows a much smaller water accessibility increase under the same pH change and Ca^2+^ concentration changes. The high‐pH NL63 ETM has similar water accessibilities as the high‐pH SARS‐CoV‐2 ETM. Thus, protons and calcium ions do not cause large expansion of the channel pore of NL63 ETM, unlike SARS‐CoV‐2 E viroporin (Figure 7c,d).

The different amino acid sequences between the NL63 and SARS ETM proteins suggest possible reasons for the distinct pH and Ca^2+^ responses of the two proteins. First, NL63 E possesses a ^7^DDN^9^ motif at the N‐terminal entrance to the TM segment, whereas the corresponding motif in SARS‐CoV ETM is ^7^EET^9^. Glu8 in SARS‐CoV‐2 E is in hydrogen‐bonding contact with Thr9 of the neighboring helix, and both residues participate in a polar network at the N‐terminal region of the SARS ETM helical bundle (Medeiros‐Silva et al., 2023). This polar network, rich in carboxyl and hydroxyl groups, is likely involved in proton binding and unbinding as well as Ca^2+^ coordination. In NL63 ETM, the shorter Asp sidechain is less likely to interact with the neighboring helix's acidic residues. Moreover, Asp sidechains have a pK_*a*_ of 3.65, whereas Glu sidechains have a pK_*a*_ of 4.25 at 25°C. Thus, the Asp‐containing NL63 ETM is expected to be more negatively charged than the Glu‐containing SARS‐CoV‐2 ETM at pH 4.5. These differences in the sidechain charge and lengths may make the ^7^DDN^9^ motif less sensitive to proton and calcium concentrations than the ^7^EET^9^ motif. Second, NL63 ETM has a long hydrophobic segment from I16 to M30, similar to SARS ETM, which has a continuous hydrophobic segment from V17 to V29. However, the SARS hydrophobic segment contains three Phe residues that are three residues apart, ^20^FxxFxxF^20^. These Phe residues are thus able to stack along the helix axis. The high‐resolution structures of SARS ETM in the closed and open states suggest that the ^20^FxxFxxF^26^ motif serves as a gate for cation conduction (Medeiros‐Silva et al., 2022; Medeiros‐Silva et al., 2023). In comparison, NL63 ETM has three consecutive Phe residues at ^24^FFF^26^, which face different orientations of the helices. As a result, these residues may be unable to perform the gating function, diminish ion conduction at low pH. Phe residues are completely absent in the 229E E protein, which has a much higher sequence homology with NL63 E than SARS E. Given that 229E and NL63 belong to the same CoV group, we speculate that the Phe residues do not play a role in the ion permeability of NL63 ETM. Future experiments would be required to elucidate the structural mechanisms of the limited NL63 ETM pore expansion at acidic pH.

In cells, the ERGIC compartment has a pH of ~6 and free Ca^2+^ levels of 200–500 μM (Appenzeller‐Herzog & Hauri, 2006; Sargeant & Hay, 2022; Zhou et al., 2009). These concentrations are lower than those in the current NMR samples. However, even under higher proton and Ca^2+^ concentrations, the current NMR data show that the NL63 ETM water accessibility does not increase substantially from the high‐pH state, implying that NL63 E may not conduct cations as efficiently as SARS E in vivo. This implication is in good agreement with the low channel activity of the 229E E protein compared to SARS‐CoV‐2 E (Wilson et al., 2006). In vivo, the NL63 virus causes bronchitis and croup, especially in young children, but symptoms are much milder than those of SARS‐CoV‐2 (Abdul‐Rasool & Fielding, 2010). We speculate that the reduced virulence of NL63 might partly result from the weaker ability of the NL63 E protein to cause ion imbalance in the host cell. Future biochemical, biophysical, and structural experiments will be of interest to test this hypothesis in detail.

## 4 MATERIALS AND METHODS

### 4.1 Cloning, expression, and purification of NL63 E proteins

We designed two constructs of the NL63 E protein: E (1–37) contains the N‐terminal ectodomain and the TM domain, while ETM (7–37) contains only the TM domain. The E (1–37) coding gene was purchased from GenScript and subcloned into a Champion pET‐SUMO plasmid. The E (7–37) construct was obtained from the E (1–37)‐encoding plasmid with the Gibson Assembly® Kit. All plasmids used in this work encode the E protein construct of interest fused with the 98‐residue SUMO solubility tag and an N‐terminal His_6_‐tag.

The two E proteins were expressed and purified as SUMO fusion proteins as described recently (Sučec et al., 2023). Briefly, we transformed the plasmids into *E. coli* BL21(DE3) cells (New England Biolabs) and grew a starter culture overnight in 50 mL of LB medium containing 50 μg/mL of kanamycin at 37°C under 220 rpm shaking. The cells were harvested by centrifugation at 1000*g* and inoculated into 1 L of M9 media to reach an optical density (OD_600_) of 0.2. The M9 media contained 5.25 g/L of Na_2_HPO_4_, 3 g/L of KH_2_PO_4_, 0.5 g/L of NaCl, 1 g/L of ^15^NH_4_Cl, 3 g/L of uniformly ^13^C‐labeled glucose, 1 mM of MgSO_4_, 0.1 mM of CaCl_2_, 50 μg/mL of kanamycin, minimum essential medium (MEM) vitamins, and mineral supplements. Cells were grown at 37°C under 200 rpm shaking until the OD_600_ reached 0.7–0.9. The culture was cooled for 30 min at 4°C, followed by induction of protein expression with 0.4 mM IPTG. Expression proceeded for 18 h at 18°C under 200 rpm shaking.

After overnight protein expression, the cells were harvested by centrifugation at 5500 g and resuspended on ice in 80 mL of lysis buffer containing 50 mM of Tris at pH 8, 100 mM of NaCl, 1 mM of MgSO_4_, and 1% Triton X‐100. To facilitate cell lysis, 20 mg of lysozyme, 1 tablet of cOmplete™ protease inhibitor cocktail, and 1 μL of Benzonase® Nuclease were added to the cell suspension prior to sonication. Cells were lysed on ice by probe sonication with 2 s “pulse on,” 20 s “pulse off,” 30% amplitude, and 5 min total “on” time. The soluble fraction was collected and purified using a Ni^2+^ affinity column, followed by reverse‐phase HPLC (Figure S5). For Ni^2+^ column purification, we added 10 mM imidazole to the soluble fraction, incubated the solution with 6 mL of Ni^2+^‐charged iminodiacetic acid resin for 1 h at 4°C with gentle rocking, then washed with 100 mL of wash buffer (50 mM Tris pH 8, 100 mM NaCl, 0.1% DDM, 20 mM imidazole) to remove non‐specifically bound proteins. The His_6_‐SUMO‐E fusion protein was eluted from the resin in two batches of 50 mL of elution buffer (50 mM Tris pH 8, 100 mM NaCl, 0.1% DDM, 250 mM imidazole). The fusion protein concentration in the eluted fractions was estimated from the absorbance at 280 nm (A_280_). Then SUMO‐protease (Ulp1) was added to the elution fractions with a 1: 1 E: Ulp1 mass ratio to cleave the SUMO tag. The cleavage reaction was supplemented with 2 mM TCEP (final concentration) and diluted with dilution buffer (50 mM Tris, pH 8, 80 mM NaCl, 0.025% DDM) to a final concentration of 0.06% DDM. The cleavage reaction proceeded for 16 h at 4°C under slow rocking.

To purify the cleaved E protein, we loaded the supernatant onto an Agilent C3 column (21.2 mm × 150 mm, 5 μm particle size) for reverse‐phase HPLC on a Varian Prostar 210 System. The column was run with a linear gradient of 5%–95% 9:1 acetonitrile/isopropanol with 0.1% trifluoroacetic acid (TFA) in Channel B with a flow rate of 10 mL/min. Channel A was water with 0.1% TFA. The purity of the proteins was assessed by MALDI‐TOF mass spectrometry (Figure S5c,f). The measured mass of E (1–37) differs from the theoretical mass by 36 Da, which may reflect oxidation of the two Met residues to methionine sulfoxide during the mass spectrometry experiment. MALDI‐TOF and solid‐state NMR spectra of ETM (7–37) show reduced Met. Purified fractions were concentrated using a rotary evaporator, then lyophilized to obtain protein powders. From 1 L of M9 medium, we obtained ~16 mg of E (1–37) and ~ 9 mg of ETM (7–37).

### 4.2 Preparation of membrane‐bound E samples

Purified E proteins were reconstituted into a lipid membrane containing 1‐palmitoyl‐2‐oleoyl‐sn‐glycero‐3‐phosphocholine (POPC), 1‐palmitoyl‐2‐oleoyl‐sn‐glycero‐3‐phospho‐ethanolamine (POPE), 1‐palmitoyl‐2‐oleoyl‐sn‐glycero‐3‐phospho‐l‐serine (POPS), and cholesterol at molar ratios of 45: 20: 20: 15. This membrane mixture is denoted as POPX/Chol below. Phospholipids (Avanti Polar Lipids) were dissolved in chloroform. The E protein was dissolved in hexafluoroisopropanol (HFIP) and diluted 10‐fold with methanol. This procedure for solubilizing NL63 E proteins is similar to the use of TFE and ethanol to reconstitute SARS‐CoV‐2 E proteins into lipid bilayers for measuring ion channel currents (Li et al., 2014; Wilson et al., 2004). The protein solution was mixed with the lipid solution at protein‐lipid molar ratios (P/L) of 1:20 for ETM (7–37) and 1:25 for E (1–37). The organic solvent was removed under a flow of nitrogen gas, then 200 μL of cyclohexane were added to the translucent film and lyophilized overnight to give a white homogeneous dry powder. The film was resuspended in 4 mL of either buffer A (25 mM Tris pH 7.4, 25 mM NaCl, 1 mM EDTA, 0.07 mM NaN_3_) or buffer B (25 mM sodium acetate pH 4.5, 25 mM Ca^2+^, 25 mM NaCl, 0.07 mM NaN_3_). The proteoliposome suspension was homogenized with three rounds of vortexing (3 s) and sonication (3 s) in a bath sonicator. The resulting suspension was incubated for 60 min at room temperature with occasional stirring to fully hydrate the proteoliposomes. Next the suspension was subjected to eight freeze–thaw cycles, alternating between freezing in liquid nitrogen and thawing at 42°C in a water bath. The vesicle solution was centrifuged at 250,000 g for 6–18 h using a Beckman SW60‐Ti swinging‐bucket rotor to obtain a wet membrane pellet, which was placed in a desiccator until it reached a hydration level of ~40% (w/w) water relative to total mass. The pellets were packed into 3.2 ‐mm magic‐angle‐spinning (MAS) rotors. Each rotor typically contained 4–5 mg of ^13^C, ^15^N‐labeled protein.

### 4.3 Solid‐state NMR experiments

All ssNMR experiments were conducted at the Francis Bitter Magnet Lab (FBML). MAS experiments were conducted on an 800 MHz (18.8 T) Bruker AVANCE spectrometer using a BlackFox 3.2 mm ^1^H/^13^C/^15^N MAS probe. MAS frequencies were 14 kHz or 10.5 kHz. ^13^C chemical shifts were externally referenced to the adamantane CH_2_ chemical shift at 38.48 ppm on the tetramethylsilane (TMS) scale. ^15^N chemical shifts were referenced to the ^15^N signal of ^15^N‐acetylvaline at 122.0 ppm on the liquid ammonia scale. ^1^H chemical shifts were referenced internally to the POPC Hγ chemical shift at 3.264 ppm on the DSS scale. The reported sample temperatures were estimated based on the ^1^H chemical shift of bulk water ($\delta_{H2O}$) using the equation $T_{\mathrm{eff}}\;\left(K\right)=96.9∙\left(7.83–\delta_{H2O}\right)$ (Böckmann et al., 2009).

2D ^13^C‐^13^C dipolar correlation spectra were measured using 25 ms of Combined R2nv‐Driven (CORD) mixing for ^13^C spin diffusion (Hou et al., 2013). 2D ^15^N‐^13^Cα correlation spectra were measured using 4.5 ms of SPECIFIC‐CP for ^15^N‐^13^C dipolar polarization transfer. Water‐ and lipid‐edited ^13^C spectra were measured at 25°C. The water or lipid ^1^H signals were first selected using a ^1^H T_2_ filter containing a 180° Gaussian pulse. For water‐edited experiments, the 180° pulse length was 2.57 ms, the total T_2_ filter time was 2.71 ms, and the ^1^H carrier frequency was 4.75 ppm. For the lipid‐edited experiments, the Gaussian 180° pulse length was 3.0 ms, the total T_2_ filter time was 3.14 ms, and the ^1^H carrier frequency was 1.3 ppm. The selected water or lipid ^1^H magnetization was transferred to protein protons using a variable mixing time of 4 to 100 ms. The intensities of the water‐edited 2D CC spectra were compared with the intensities of the unedited spectra. The detailed parameters of the NMR experiments are listed in Table S7.

All NMR spectra of NL63 ETM were measured at a sample temperature of 25°C, and the spectra of E(1–37) were measured at 22°C. During the initial optimization of the 2D experiments, we measured the 2D NCA spectra of ETM at 25°C and 10°C and found slightly narrower linewidths at 25°C. Thus, we measured subsequent spectra at 25°C. The line broadening at lower temperatures is consistent with the immobilization of multiple conformations of the protein, giving different chemical shifts.

### 4.4 Chemical shift assignment from 2D and 3D correlation spectra

We assigned ^13^C and ^15^N chemical shifts of the rigid residues of the two NL63 E constructs at pH 7.4 using 2D ^13^C‐^13^C (CC), ^15^N‐^13^Cα (NC) and 3D NCACX, NCOCX, CONCA spectra. The NCACX spectra provide intra‐residue ^15^N and ^13^C chemical shifts, while the NCOCX spectra provide sequential correlations between ^15^N of residue *i* and ^13^C of residue *i*‐1. The CONCA spectra provide sequential correlations between ^15^N and ^13^Cα of residue *i* and the ^13^C’ chemical shift of residue *i*‐1.

^13^C and ^15^N chemical shifts of the low‐pH ETM sample were transferred from the assignment of the pH 7.4 ETM sample. The combined ^13^Cα and ^15^N chemical shift perturbations of the ETM sample between the low and high pH were calculated using the equation $\mathrm{CSP}={\left(0.3{{\Delta \delta }_{13C}}^{2}+0.14{{\Delta \delta }_{15N}}^{2}\right)}^{1/2}$, where the scaling factors account for the different gyromagnetic ratios of ^13^C and ^15^N (Williamson, 2013).

Backbone (ϕ, ψ) torsional angles of the two NL63 E constructs at pH 7.4 and ETM at pH 4.5 in the presence of Ca^2+^ were predicted using the TALOS‐N software (Shen & Bax, 2013). All assigned chemical shifts were used as the input.

### 4.5 Analysis of water‐edited ^13^C‐^13^C and ^15^N‐^13^C correlation spectra

To quantify the water accessibility of NL63 ETM with high sensitivity, we measured water‐edited 2D CC and 2D NC spectra with 9 ms water spin diffusion (*S* (2D, 9 ms)) and control spectra without water‐editing ($S_{0}\left(2D\;\text{unedited}\right)$). We used these spectra to indirectly calculate the water‐transferred intensity ratios between the 9 ms and 100 ms water‐transferred spectra. To calculate these ratios for both 2D CC and 2D NC spectra, we used a custom‐written Python script that extracts the intensities of the $S\left(2D,9\;\mathrm{ms}\right)$ and $S_{0}\left(2D\;\text{unedited}\right)$ spectra using the *nmrglue* package (Helmus & Jaroniec, 2013). These 2D intensity ratios were then scaled by the intensity ratio between the unedited 1D spectrum, $S\left(1D,\text{unedited}\right)$ and the 1D 100 ms water‐edited spectrum, $S\left(1D,100\;\mathrm{ms}\right)$, according to the equation:

$$\frac{S\left(2D,9\;\mathrm{ms}\right)}{S_{0}\left(2D,100\;\mathrm{ms}\right)}=\frac{S\left(2D,9\;\mathrm{ms}\right)}{S_{0}\left(2D\;\text{unedited}\right)}∙\frac{S\left(1D,\text{unedited}\right)}{S_{0}\left(1D,100\;\mathrm{ms}\;\right)}$$

For water‐edited 2D CC data, the 100 ms water‐edited 1D ^13^C CP and unedited ^13^C CP spectra were used as the $S\left(1D,100\;\mathrm{ms}\right)$ and $S\left(1D,\text{unedited}\right)$. For water‐edited 2D NC data, the 100 ms water‐edited 1D NCA‐filtered ^13^C spectra and the unedited 1D NCA‐filtered ^13^C spectra were used as the $S\left(1D,100\;\mathrm{ms}\right)$ and $S\left(1D,\text{unedited}\right)$. The 100 ms water‐edited ^13^C spectral intensities are 17–20% of the unedited ^13^C CP intensity.

2D contour maps of the water‐edited 2D CC spectra (Figure 6) were generated using another custom‐written Python script. To avoid division artifacts, we set all intensities below a threshold value in the 9 ms *S* spectrum to zero. This threshold is 3.5 times the standard deviation of noise for the pH 7.4 spectrum and 4.2 times the standard deviation of noise for the pH 4.5 Ca^2+^‐bound spectrum. For the control 2D CC spectrum, we set to −1 all intensities that are 7 times below the standard deviation of noise. Only positive intensity ratios are plotted in the hydration map.

Residue‐specific water accessibility values S/S_0_ were extracted from the intensity ratios *S*(2D, 9 ms)/*S*_0_ (2D, 100 ms) from water‐edited 2D CC (Figure 6) and NC spectra (Figure S3) using another custom‐written Python script. For water‐edited 2D CC spectra, we extracted the values for selected cross‐peaks for each residue, then averaged them to give the final residue‐specific water accessibilities. The error bars of the averaged values were propagated as ${\bar{\left(∆\right)}}_{k}=\sqrt{\sum_{i}{{∆}^{i}}_{k}}/N$, where ${{∆}^{i}}_{k}$ is the error bar for the water accessibility of the *i*‐th cross‐peak of the *k*‐th residue, propagated from the spectral noise, and N is the number of cross‐peaks extracted for each residue. For residue‐specific S/S_0_ from the 2D NC spectra, no averaging was applied as each residue is represented by a single ^15^N‐^13^Cα cross peak.

### 4.6 Analysis of leu, val, ile intensities from water‐edited 2D CC spectra

We applied a recently introduced methyl NMR analysis to obtain information about the helix packing of NL63 ETM from water‐edited 2D CC spectra (Sučec et al., 2023). The intensities of all Cβ cross‐peaks of Leu, Val, and Ile residues of the 2D spectra were integrated in the TopSpin software. Identical integration regions and identical line broadening parameters lb = −20 and gb = 0.03 were used for the non‐edited and water‐edited 2D CC spectra. The Cβ cross‐peak intensities in the non‐edited spectrum were summed, then normalized to the number of Leu residues, which is 6 in ETM and 8 in E (1–37). Due to different polarization transfer (PT) efficiencies of the three amino acids, we applied correction factors to Val and Ile to match the numbers of these residues in the protein. Subsequently, the same PT correction factors were applied to the intensities of the water‐edited spectra. These methyl cross peak intensities were then compared with the numbers of water‐accessible Leu, Val, and Ile residues in seven helical packing models. The seven helix‐packing topologies, representing an oligomeric helical bundle formed by ideal heptad repeats, are denoted by the heptad positions of residue Asn14. To count the number of water accessible residues in different models, we consider the pore‐facing (*a*, *d*) residues to be 100% water accessible and the interfacial (*e.g.*) residues as 50% water.

## AUTHOR CONTRIBUTIONS

**Mei Hong:** Conceptualization; formal analysis; writing – review and editing; supervision; resources; funding acquisition. **Iva Sučec:** Data curation; formal analysis; writing – original draft; investigation; writing – review and editing. **Yanina Pankratova:** Formal analysis; writing – original draft; writing – review and editing. **Mriganka Parasar:** Data curation.

## CONFLICT OF INTEREST STATEMENT

The authors declare no potential conflict of interest.

## Supporting information


**Data S1.** Supporting Information.
