# Supplementary material for: Transmembrane conformation of the envelope protein of an alpha coronavirus, NL63
Source: Protein Sci. 2024 Mar 19;33(4):e4923. doi: 10.1002/pro.4923 (PMC10949323; doi:10.1002/pro.4923)
Supplement: Supplementary file 1 — Data S1. Supporting Information. [file PRO-33-e4923-s001.pdf]

# Supporting Information

## Transmembrane Conformation of the Envelope Protein of an Alpha Coronavirus, NL63

Iva Sučec, Yanina Pankratova, Mriganka Parasar and Mei Hong*

Department of Chemistry, Massachusetts Institute of Technology, 170 Albany Street, Cambridge, MA 02139

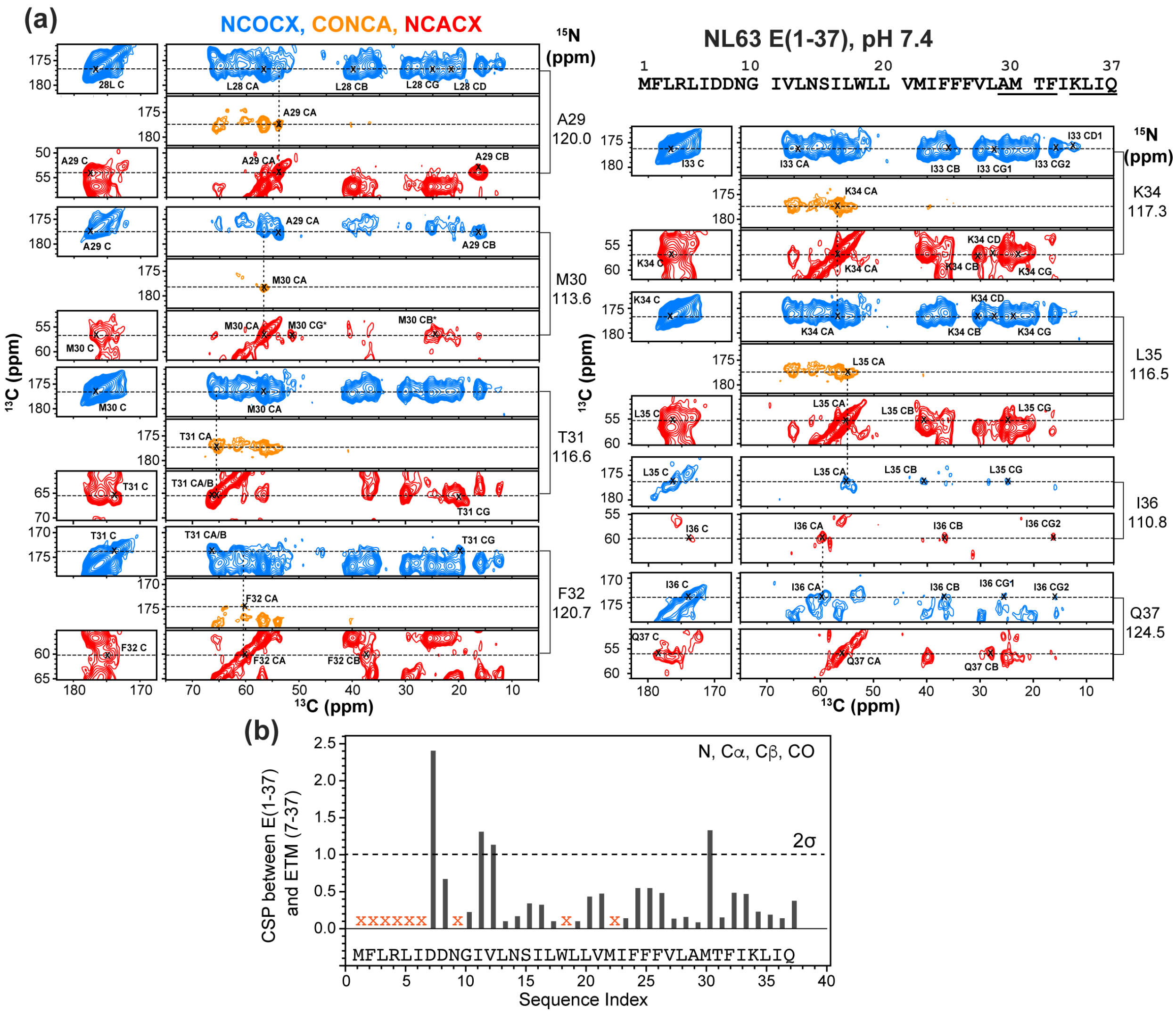

**Figure S1**. 3D spectral strips of NL63 E (1-37) for resonance assignment and chemical shift differences between ETM (7-37) and E (1-37). (**a**) 3D NCOCX, CONCA and NCACX spectral strips of membrane-bound E (1-37) at pH 7.4 for residues A29 to Q37 (underlined in the amino acid sequence). M30 Cβ and Cγ chemical shifts in NCACX spectra (denoted as an asterisk) indicate oxidation of this residue in E (1-37). (**b**) Chemical shift difference between ETM (7-37) and E (1-37) at pH 7.4. The backbone $^{15}$N, $^{13}$Cα, $^{13}$Cβ and $^{13}$CO chemical shifts are included (**Table S5**). Chemical shift differences below 1.01 ppm (2σ) are considered insignificant. Residues that are unassigned in at least one of the two constructs are marked with orange 'x'.

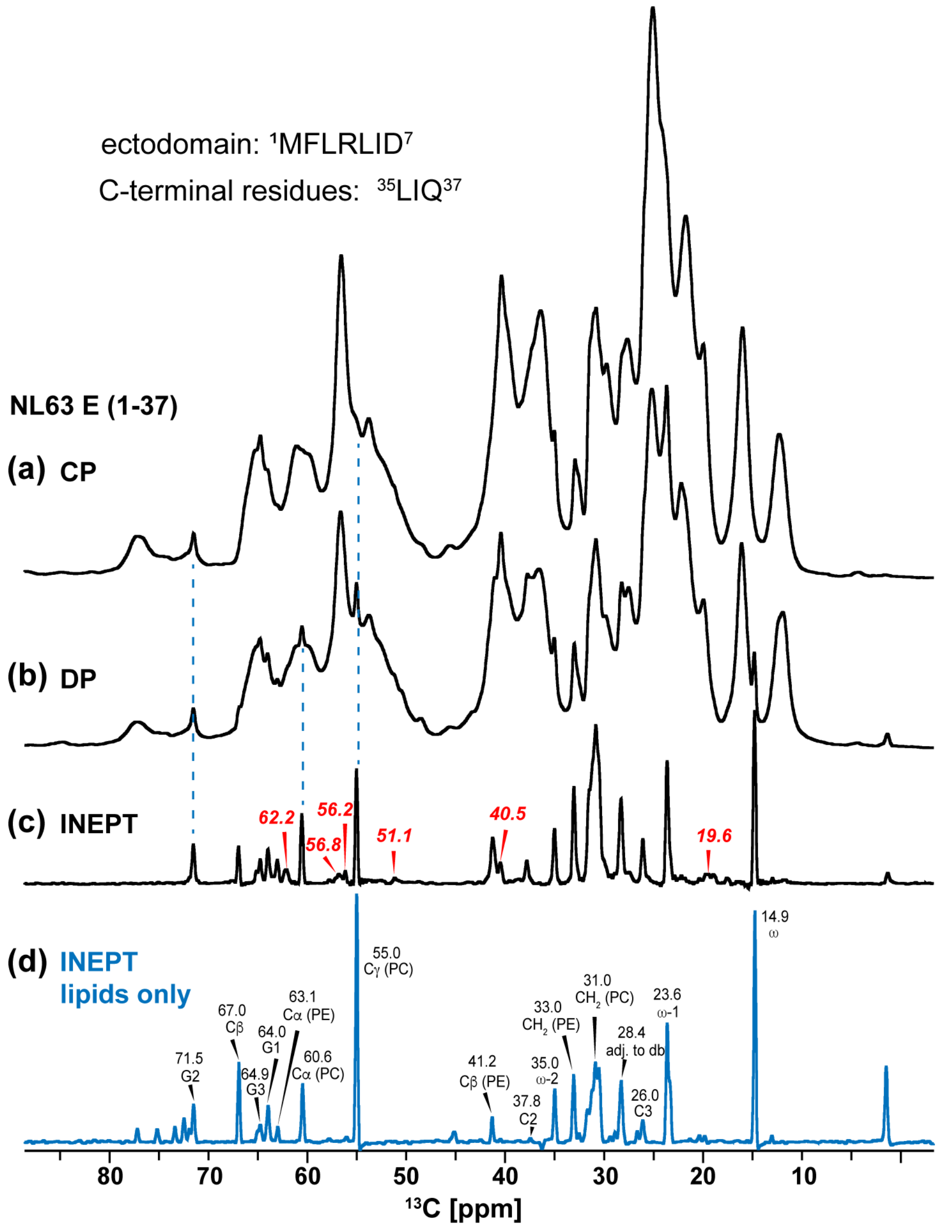


**Figure S2**. $^{13}$C 1D spectra of NL63 E (1-37) in the POPX/cholesterol membrane (**a-c**) and a membrane-only sample (**d**). The spectra were measured at 22°C. (**a**) Cross-polarization (CP) spectrum of membrane-bound E (1-37) preferentially detects immobilized residues and lipid segments. (**b**) Direct polarization (DP) spectrum of membrane-bound E (1-37) detects the signals of both immobilized and dynamic species. Dashed lines guide the eye for some of the sharp lipid signals that overlap with the broad peptide signals. (**c**) INEPT spectrum of membrane-bound E (1-37). Due to magnetization transfer by weak $^{1}$H-$^{13}$C scalar couplings, only highly mobile segments with long $^{1}$H and $^{13}$C $T_2$ relaxation times are detected. (**d**) $^{13}$C INEPT spectrum of a protein-free ERGIC membrane that contains POPC, POPE, POPS, PI and cholesterol. The lipid assignments are shown. This control spectrum allows the identification of several weak protein signals in (**c**), whose chemical shifts (indicated in red) are tentatively attributed to Met, Leu and Ile residues in the ectodomain and/or the C-terminal three residues of the protein.

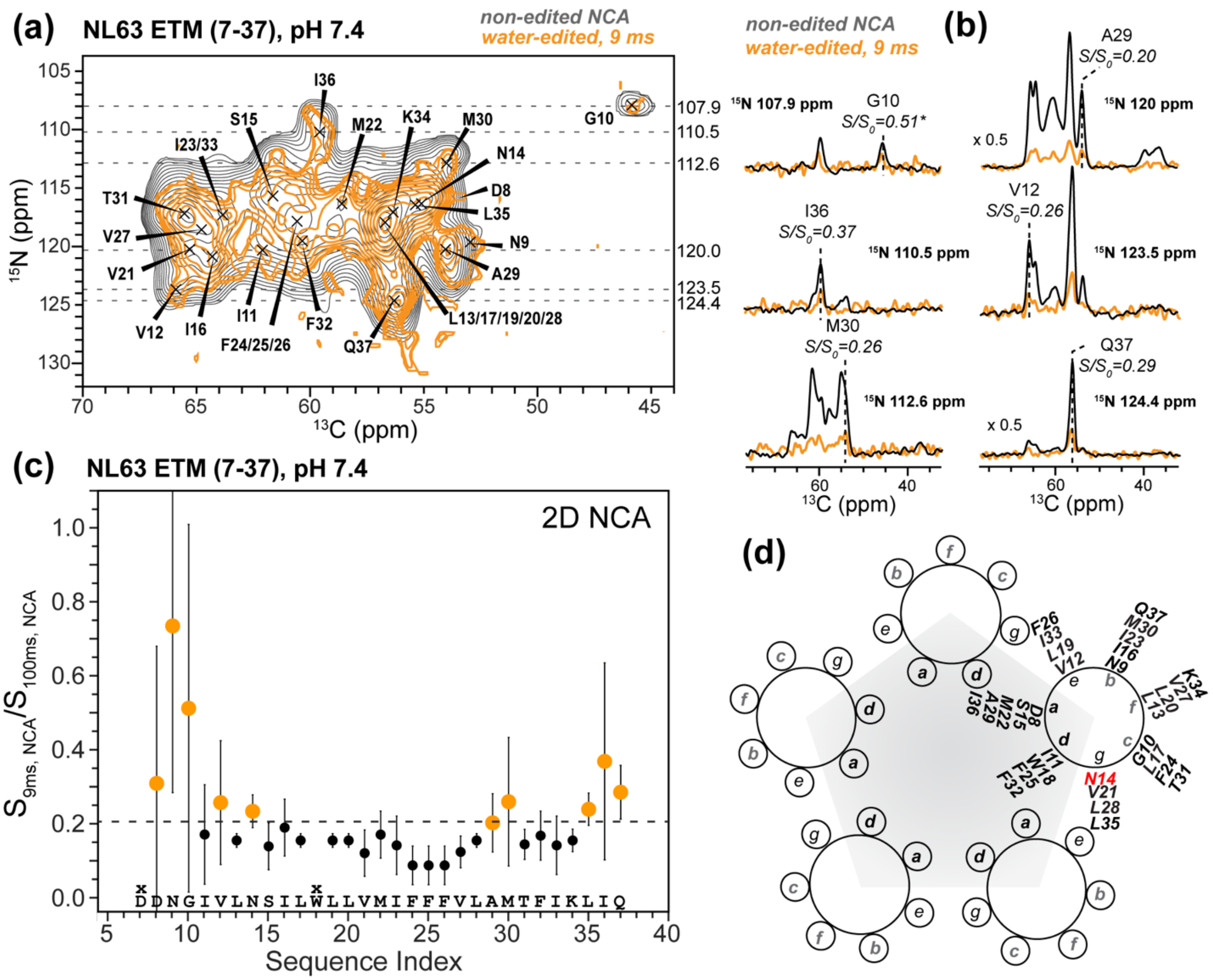


**Figure S3.** Water-edited 2D NCA spectra of membrane-bound NL63 ETM (7-37) at pH 7.4. (**a**) Water-edited 2D NCA spectrum measured with 9 ms $^{1}$H spin diffusion (orange), overlaid with the unedited 2D NCA spectrum (grey). (**b**) Representative $^{15}$N cross sections of the 2D spectra. An intensity factor of 0.5 was applied to the 120.0 ppm and 124.4 ppm $^{15}$N cross sections for clarity. **(c)** Residue-specific water accessibility from the intensity ratios of the 9 ms and 100 ms water-edited NCA spectra. Error bars were propagated from spectral noise. Unassigned residues are indicated with 'x' symbols. Dashed line indicates average water accessibility. $S/S_0$ values that are higher than the average are shown in orange. **(d)** Schematic of the ETM pentamer topology with residue N14 at heptad position *g*.

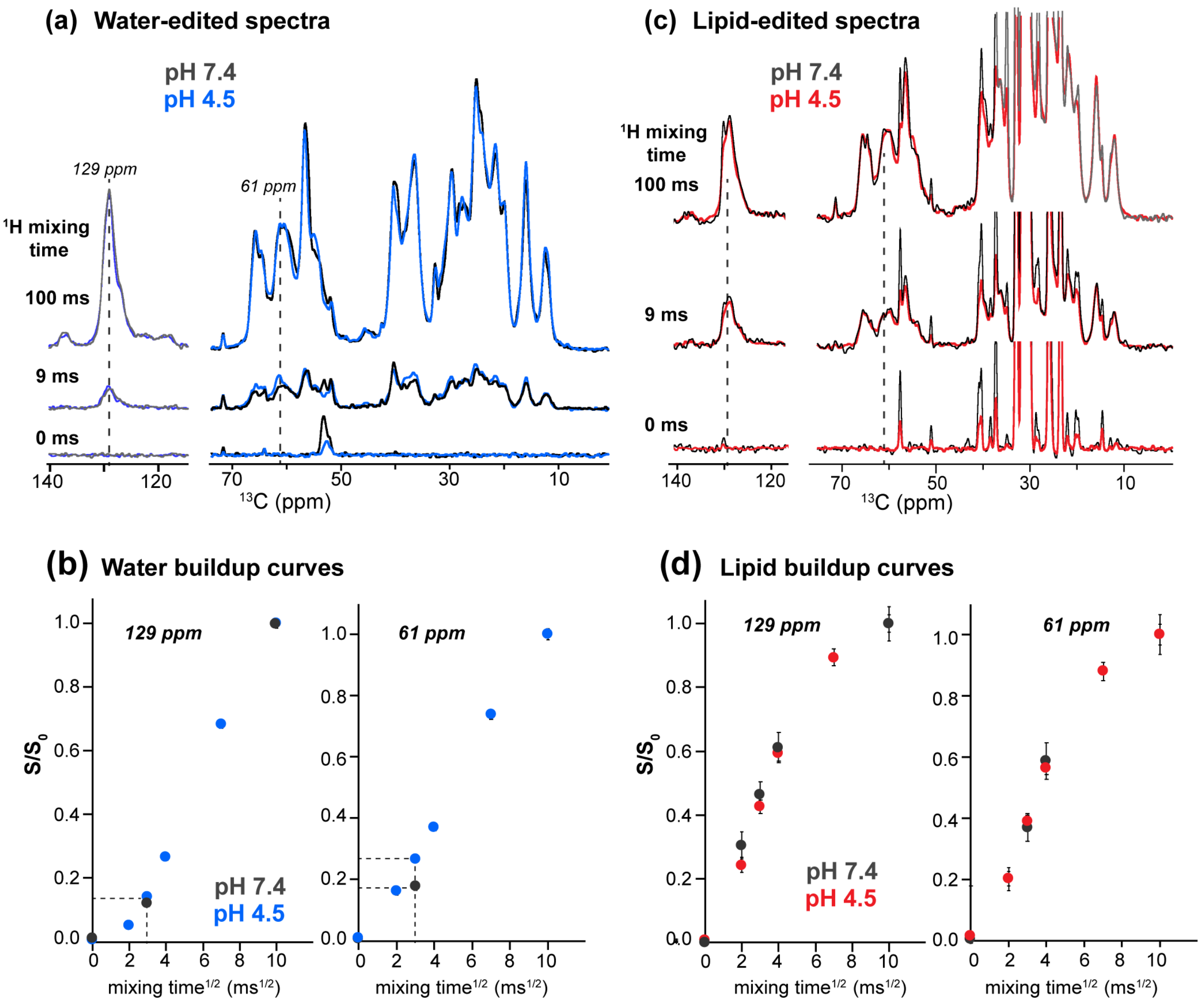


**Figure S4**. Water and lipid $^{1}$H spin diffusion buildup rates of NL63 ETM(7-37) at pH 7.4 (grey spectra) versus at pH 4.5 with $Ca^{2+}$ bound (blue and red spectra). (**a**) 1D water-edited $^{13}$C spectra of the protein with $^{1}$H mixing times of 0, 9 and 100 ms. (**b**) Water buildup curves for the 129-ppm peak and the 61-ppm peak. The low-pH $Ca^{2+}$ bound sample has slightly faster water magnetization transfer than the neutral pH sample at short mixing times. (**c**) 1D lipid-edited $^{13}$C spectra of the protein with $^{1}$H mixing times of 0, 9, and 100 ms. (**d**) Lipid buildup curves extracted at 129 ppm and 61 ppm. The low-pH $Ca^{2+}$-bound sample has similar lipid $^{1}$H magnetization transfer rates as the high-pH sample.

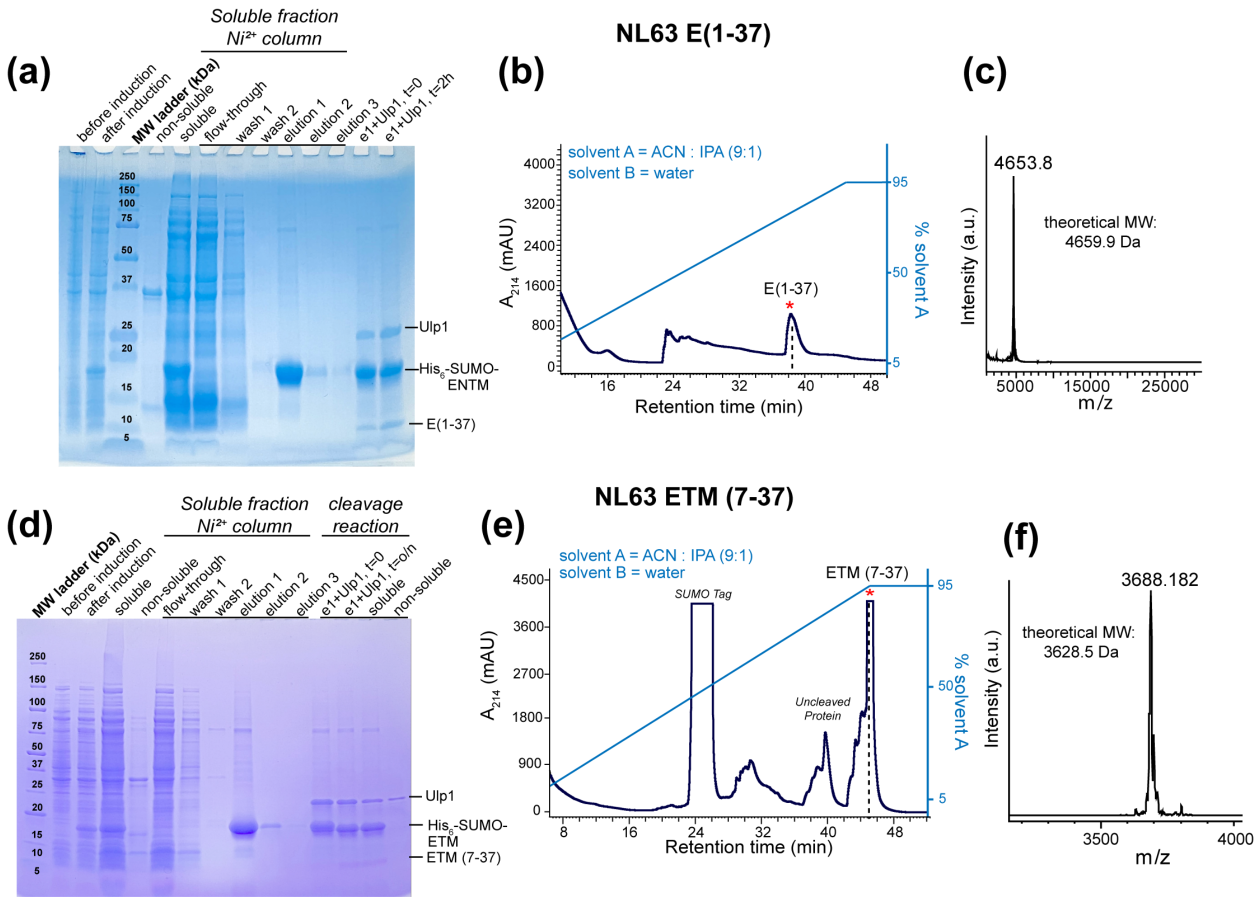


**Figure S5**. Expression and purification of NL63 E (1-37) (**a-c**) and ETM (7-37) (**d-f**). (**a, d**) SDS-PAGE of the two proteins. (**b, e**) HPLC chromatographs after cleavage of the SUMO tag. Both constructs were purified using acetonitrile/IPA as the mobile phase. E (1-37) eluted at 38.5 minutes while ETM (7-37) eluted at 45 minutes. (**c, f**) MALDI mass spectra of the proteins after HPLC, extracted at the positions indicated by the dashed lines in the HPLC chromatographs.

**Table S1.** $^{13}$C and $^{15}$N chemical shifts (ppm) of membrane-bound NL63 E (1-37) at pH 7.5. Italics indicate ambiguous assignments.

| | **HN** | **CO** | **Cα** | **Cβ** | **Cγ/Cγ1** | **Cγ2** | **Cδ/Cδ1** | **Cε** | **Cζ** | **Nζ** | **Nε** |
|---|---|---|---|---|---|---|---|---|---|---|---|
| **1M** | | | | | | | | | | | |
| **2F** | | | | | | | | | | | |
| **3L** | | | | | | | | | | | |
| ***4R*** | *122.6* | | *52.7* | *25.4* | *25.4* | | | | *158* | | |
| **5L** | | | | | | | | | | | |
| **6I** | | | 57.6 | 36.7 | 27.6 | 16.5 | 13.4 | | | | |
| ***7D*** | | | *52.1* | *41.9* | | | | | | | |
| ***8D*** | | | *53.6* | *40.6* | | | | | | | |
| **9N** | | | | | | | | | | | |
| **10G** | 106.9 | 174.3 | 45.74 | | | | | | | | |
| **11I** | 121.6 | 175.9 | 64.1 | 36.3 | 28 | 15.7 | 12.4 | | | | |
| **12V** | 118.8 | 176 | 65.3 | 29.8 | 21.5 | | | | | | |
| ***13L*** | *118.1* | *176.8* | *56.8* | *40* | *25.2* | | *21.8* | | | | |
| **14N** | 116.4 | 176.3 | 54.9 | 36.7 | 173.6 | | | | | | |
| **15S** | 115.8 | 174.2 | 61.1 | 61.86 | | | | | | | |
| **16I** | 122 | 175.7 | 64.4 | 36.1 | 28.2 | 15.6 | 12.8 | | | | |
| ***17L*** | *119.1* | *176.6* | *56.8* | *39.9* | *25.3* | | *21.9* | | | | |
| **18W** | | | | | | | | | | | |
| ***19L*** | | *176.6* | *56.8* | *39.9* | *25.3* | | *21.9* | | | | |
| ***20L*** | | *176.6* | *56.8* | *39.9* | *25.3* | | *21.9* | | | | |
| ***21V*** | *117.8* | *176* | *65.3* | *29.8* | *21.5* | | | | | | |
| **22M** | | | | | | | | | | | |
| ***23I*** | *118.3* | *176* | *64.6* | *36.5* | *28* | *16.06* | *12.4* | | | | |
| ***24F*** | *119.5* | *176* | *60.2* | *37.4* | | | | | | | |
| ***25F*** | *119.5* | *176* | *60.2* | *37.4* | | | | | | | |
| ***26F*** | *119.5* | *176* | *60.7* | *37.4* | | | | | | | |
| **27V** | 118 | 176.1 | 65.4 | 29.9 | 21.7 | | | | | | |
| **28L** | 116.8 | 176.7 | 56.8 | 40 | 25.1 | | 21.6 | | | | |
| **29A** | 120 | 177.5 | 54 | 16.5 | | | | | | | |
| **30M** | 113.6 | 176.6 | 56.7 | 25.0 | 51.6 | | | | | | |
| **31T** | 116.6 | 173.8 | 65.5 | 66.4 | 19.8 | | | | | | |
| **32F** | 120.7 | 175.5 | 60.2 | 37.4 | | | | | | | |
| ***33I*** | | *176.7* | *64.3* | *36.3* | *27.9* | *15.9* | *12.2* | | | | |
| **34K** | 117.3 | 176.7 | 56.9 | 30.6 | 23.9 | | 27.5 | | | 32.09 | |
| **35L** | 116.5 | 176.3 | 55.3 | 40.7 | 24.9 | | | | | | |
| **36I** | 110.8 | 173.9 | 59.8 | 36.8 | 25.6 | 16.4 | 12.7 | | | | |
| **37Q** | 124.5 | 178.4 | 56 | 28.1 | 32.8 | | 179.2 | | | | 111.3 |

**Table S2.** $^{13}C$ and $^{15}N$ chemical shifts (ppm) of membrane-bound NL63 ETM (7-37) at pH 7.5 and at pH 4.5 with $Ca^{2+}$. Italics indicate ambiguous assignments.

| residue | pH | HN | CO | Cα | Cβ | Cγ/Cγ1 | Cγ2 | Cδ/Cδ1 | Cε | Nδ/Nζ | Nε |
|---|---|---|---|---|---|---|---|---|---|---|---|
| **7D** | 7.5 | | 170.6 | 51.6 | 38.2 | | | | | | |
| **7D** | 4.5 | | 171.1 | 51.5 | 37.8 | | | | | | |
| **8D** | 7.5 | 119.3 | 175.0 | 53.4 | 39.3 | | | | | | |
| **8D** | 4.5 | 118.9 | | 53.9 | 39.5 | | | | | | |
| **9N** | 7.5 | 120.4 | 174.5 | 52.2 | 36.9 | | | | | | |
| **9N** | 4.5 | 120.9 | 174.5 | 52.6 | 36.6 | | | | | | |
| **10G** | 7.5 | 109.6 | 174.0 | 45.7 | | | | | | | |
| **10G** | 4.5 | 109.2 | 173.8 | 45.8 | | | | | | | |
| **11I** | 7.5 | 119.4 | 177.0 | 62.9 | 35.7 | 27.1 | 11.6 | 15.8 | | | |
| **11I** | 4.5 | 120.4 | | 63.1 | 35.6 | 27.2 | 11.8 | 15.7 | | | |
| **12V** | 7.5 | 124.6 | 176.0 | 65.7 | 29.4 | 21.4 | 19.6 | | | | |
| **12V** | 4.5 | 123.7 | | 65.6 | 29.4 | 21.3 | 19.6 | | | | |
| ***13L*** | 7.5 | | *176.7* | *56.7* | *39.9* | *25.2* | | | | | |
| **13L** | 4.5 | | | | | | | | | | |
| **14N** | 7.5 | 117.1 | 176.1 | 54.5 | 36.1 | 173.9 | | | | 110.1 | |
| **14N** | 4.5 | 116.9 | | 54.4 | 36.4 | | | | | | |
| **15S** | 7.5 | 117.3 | 173.8 | 61.7 | 61.9 | | | | | | |
| **15S** | 4.5 | 117.0 | | 61.4 | | | | | | | |
| **16I** | 7.5 | 122.9 | 175.5 | 64.3 | 36.2 | 29.0 | 15.6 | 12.8 | | | |
| **16I** | 4.5 | 122.7 | | 64.4 | | | | | | | |
| ***17L*** | 7.5 | | *176.7* | *56.7* | *39.9* | *25.2* | | | | | |
| **17L** | 4.5 | | | | | | | | | | |
| **18W** | 7.5 | | | 58.9 | 27.0 | 111.3 | | | | | |
| **18W** | 4.5 | | | | 26.8 | 111.5 | | | | | |
| ***19L*** | 7.5 | *123.6* | *176.7* | *56.5* | *39.5* | *25.0* | | *21.1* | | | |
| **19L** | 4.5 | *123.2* | | 56.4 | 39.6 | 25.0 | | 21.3 | | | |
| ***20L*** | 7.5 | | *177.3* | *56.5* | *39.5* | *25.0* | | *21.1* | | | |
| **20L** | 4.5 | | | | | | | | | | |
| **21V** | 7.5 | 121.4 | 176.1 | 65.4 | 29.7 | 20.3 | 21.8 | | | | |
| **21V** | 4.5 | 121.4 | | 65.6 | | | | | | | |
| **22M** | 7.5 | 117.8 | 176.6 | 58.3 | 31.2 | | | | | | |
| **22M** | 4.5 | 117.8 | | 58.7 | 31.0 | | | | | | |
| **23I** | 7.5 | 118.0 | 175.9 | 64.0 | 36.0 | 28.2 | 15.5 | 12.5 | | | |
| **23I** | 4.5 | 118.0 | | 64.1 | 36.0 | 22.0 | | 12.4 | | | |
| ***24F*** | 7.5 | *119.2* | *175.6* | *60.3* | *37.2* | *136.5* | | *129.3* | | | |
| **24F** | 4.5 | 119.1 | | 60.1 | 37.3 | 136.8 | | 129.1 | | | |
| ***25F*** | 7.5 | *119.2* | *175.6* | *60.3* | *37.2* | *136.5* | | *129.3* | | | |
| **25F** | 4.5 | | | | | | | | | | |
| ***26F*** | 7.5 | *119.2* | *175.6* | *60.3* | *37.2* | *136.5* | | *129.3* | | | |
| **26F** | 4.5 | | | | | | | | | | |
| **27V** | 7.5 | 119.4 | 176.1 | 65.0 | 29.8 | 20.2 | 21.7 | | | | |
| **27V** | 4.5 | 119.3 | | 65.2 | | | | | | | |
| **28L** | 7.5 | | 176.5 | 56.7 | 40.0 | 25.1 | | 21.4 | | | |
| **28L** | 4.5 | | | | | | | | | | |
| **29A** | 7.5 | 121.7 | 177.4 | 53.8 | 16.5 | | | | | | |
| **29A** | 4.5 | 121.7 | | 53.9 | 16.1 | | | | | | |
| **30M** | 7.5 | 113.7 | 176.8 | 53.9 | 30.5 | | | | | | |
| **30M** | 4.5 | 113.6 | | 53.7 | 30.3 | | | | | | |
| **31T** | 7.5 | 118.8 | 173.8 | 65.6 | 66.6 | 19.6 | | | | | |
| **31T** | 4.5 | 118.0 | | 65.8 | 66.4 | 19.4 | | | | | |

| | | | | | | | | | | | |
|---|---|---|---|---|---|---|---|---|---|---|---|
| **32F** | 7.5 | 121.2 | 175.7 | 60.4 | 37.4 | | | 129.5 | | | |
| **32F** | 4.5 | 120.3 | | 60.1 | | | | | | | |
| **33I** | 7.5 | 116.4 | 175.9 | 64.2 | 36.4 | 27.9 | 15.8 | 12.4 | | | |
| **33I** | 4.5 | | | | | | | | | | |
| **34K** | 7.5 | 119.0 | 176.6 | 56.5 | 29.9 | 24.0 | | 26.6 | 40.3 | 39.6 | |
| **34K** | 4.5 | 119.2 | | 56.7 | 30.2 | | | 26.4 | 40.1 | | |
| **35L** | 7.5 | 117.9 | 176.4 | 54.8 | 40.8 | 24.8 | | 21.6 | | | |
| **35L** | 4.5 | 117.8 | 177.1 | 55.0 | 40.6 | 24.7 | | 21.6 | | | |
| **36I** | 7.5 | 111.5 | 173.7 | 59.4 | 36.7 | 25.5 | 15.9 | 12.8 | | | |
| **36I** | 4.5 | 112.6 | | 59.5 | 36.5 | 25.6 | 16.1 | | | | |
| **37Q** | 7.5 | 126.3 | 179.0 | 55.6 | 28.0 | 32.5 | | 178.6 | | | 110.2 |
| **37Q** | 4.5 | 124.5 | | 55.3 | 27.7 | 32.3 | | 178.6 | | | |

**Table S3.** Chemical shift perturbations (CSPs) between E (1-37) and ETM (7-37) at pH 7.4.

| Residue | CSP (ppm) | Atom types | | Residue | CSP (ppm) | Atom types |
|---|---|---|---|---|---|---|
| M1 | - | - | | L20 | 0.43 | Ca, Cb, C |
| F2 | - | - | | V21 | 0.47 | N, Ca, Cb, C |
| L3 | - | - | | M22 | - | - |
| R4 | - | - | | I23 | 0.14 | N, Ca, Cb, C |
| L5 | - | - | | F24 | 0.55 | N, Ca, Cb, C |
| I6 | - | - | | F25 | 0.55 | N, Ca, Cb, C |
| D7 | 2.41 | Ca, Cb | | F26 | 0.48 | N, Ca, Cb, C |
| D8 | 0.67 | Ca, Cb | | V27 | 0.14 | N, Ca, Cb |
| N9 | - | - | | L28 | 0.16 | Ca, C |
| G10 | 0.22 | N, Ca, C | | A29 | 0.09 | N, Cb, C |
| I11 | 1.31 | N, Ca, C | | M30 | 1.33 | N, Ca, C |
| V12 | 1.13 | N, Ca, Cb | | T31 | 0.15 | N, Ca, Cb |
| L13 | 0.10 | Ca, Cb, C | | F32 | 0.49 | N, Ca, C |
| N14 | 0.17 | N, Cb, C | | I33 | 0.47 | Ca, Cb, C |
| S15 | 0.34 | N, Ca, Cb, C | | K34 | 0.23 | N, Ca, Cb, C |
| I16 | 0.32 | N, Cb, C | | L35 | 0.19 | N, Ca, Cb, C |
| L17 | 0.10 | Ca, C | | I36 | 0.14 | N, Ca, Cb, C |
| W18 | - | - | | Q37 | 0.38 | N, Ca, Cb, C |
| L19 | 0.10 | Ca, C | | | | |

**Table S4.** Measured and predicted numbers of pore-facing Leu, Val, and Ile residues in NL63 ETM (7-37) and E (1-37) from methyl $^{13}C$ water-CC. The measured intensities are in best agreement with the N14*d* or N14*g* topology.

| | pH 7.4 | | | | pH 4.5 | | | |
|---|---|---|---|---|---|---|---|---|
| **ETM (7-37)** | **Leu** | **Val** | **Ile** | **RMSD** | **Leu** | **Val** | **Ile** | **RMSD** |
| # of residues | 6 | 3 | 5 | | 6 | 3 | 5 | |
| 2D CC intensity (Leu=6) | 6.0 | 3.1 | 9.0 | | 6.0 | 3.3 | 9.6 | |
| PT corrected | 1.0 | 1.0 | 0.6 | | 1.0 | 0.9 | 0.5 | |
| | **Water-edited intensities** | | | | | | | |
| Measured | **0.75** | **0.35** | **0.64** | | **0.84** | **0.48** | **0.67** | |
| Predicted N14a | 4.0 | 1.5 | 0.5 | 1.99 | 4.0 | 1.5 | 0.5 | 1.92 |
| Predicted N14b | 3.0 | 1.5 | 2.5 | 1.81 | 3.0 | 1.5 | 2.5 | 1.74 |
| Predicted N14c | 1.0 | 1.5 | 3.5 | 1.79 | 1.0 | 1.5 | 3.5 | 1.74 |
| Predicted N14d | 2.5 | 1.0 | 1.5 | **1.19** | 2.5 | 1.0 | 1.5 | **1.11** |
| Predicted N14e | 4.0 | 1.5 | 1.0 | 2.00 | 4.0 | 1.5 | 1.0 | 1.92 |
| Predicted N14f | 2.0 | 1.5 | 3.5 | 1.92 | 2.0 | 1.5 | 3.5 | 1.86 |
| Predicted N14g | 1.5 | 1.0 | 2.5 | **1.22** | 1.5 | 1.0 | 2.5 | **1.16** |

| | pH 7.4 | | | |
|---|---|---|---|---|
| **E (1-37)** | **Leu** | **Val** | **Ile** | **RMSD** |
| #of residues | 3 | 3 | 6 | |
| 2D CC intensity (Leu=6) | 8.0 | 2.8 | 9.4 | |
| PT corrected | 1.0 | 1.1 | 0.6 | |
| | **Water-edited intensities** | | | |
| Measured | **1.14** | **0.46** | **0.79** | |
| Predicted N14a | 6.0 | 1.5 | 1.5 | 2.90 |
| Predicted N14b | 5.0 | 1.5 | 3.5 | 2.79 |
| Predicted N14c | 3.0 | 1.0 | 4.5 | 2.41 |
| Predicted N14d | 4.5 | 1.0 | 2.5 | **2.20** |
| Predicted N14e | 6.0 | 1.5 | 2.0 | 2.95 |
| Predicted N14f | 4.0 | 1.5 | 4.5 | 2.77 |
| Predicted N14g | 3.5 | 1.0 | 3.5 | **2.10** |

**Table S5**. Water-accessibility $S/S_0$ values (9 ms versus 100 ms) of NL63 ETM at high and low pH from water-edited 2D CC spectra.

| Residue | $S/S_0$ | error | Cross peaks used for averaging, in the order of 'w1-w2' |
|---|---|---|---|
| **pH 7.4** | | | |
| 7 | 1.00 | 1.15 | CA-C |
| 8 | - | - | none |
| 9 | - | - | none |
| 10 | 0.19 | 0.07 | CA-CA, CA-C |
| 11 | 0.16 | 0.03 | CA-CB, CB-CA, CB-CG1, CB-CD1, CG1-CB, CG1-CG2, CD1-CB |
| 12 | 0.13 | 0.02 | CA-CB, CA-CG1, CA-CG2, CB-CA, CB-CG1, CB-CG2, CG1-CA, CG1-CB, CG1-CG2, CG2-CA, CG2-CB, CG2-CG1 |
| 13 | 0.14 | 0.02 | CA-CB, CA-CG, CB-CA, CB-CG, CG-CA, CG-CB |
| 14 | 0.20 | 0.17 | CB-CA |
| 15 | 0.20 | 0.01 | CA-CB, CB-CA |
| 16 | 0.20 | 0.05 | CA-CB, CA-CG1, CA-CG2, CB-CA, CB-CG1, CB-CG2, CG1-CB, CG1-CD1, CG2-CB, CD1-CB |
| 17 | 0.14 | 0.02 | CA-CB, CA-CG, CB-CA, CB-CG, CG-CA, CG-CB |
| 18 | - | - | none |
| 19 | 0.14 | 0.02 | CA-CB, CA-CG, CB-CA, CB-CG, CG-CA, CG-CB |
| 20 | 0.14 | 0.02 | CA-CB, CA-CG, CB-CA, CB-CG, CG-CA, CG-CB |
| 21 | 0.13 | 0.02 | CA-CB, CA-CG1, CA-CG2, CB-CA, CB-CG1, CB-CG2, CG1-CA, CG1-CB, CG1-CG2, CG2-CA, CG2-CB, CG2-CG1 |
| 22 | 0.27 | 0.21 | CA-CB |
| 23 | 0.14 | 0.03 | CA-CB, CB-CA, CB-CG1, CB-CG2, CG1-CB, CG1-CD1, CG2-CB, CD1-CB, CD1-CG1 |
| 24 | 0.13 | 0.04 | CA-CB, CA-CD, CB-CA |
| 25 | 0.13 | 0.04 | CA-CB, CA-CD, CB-CA |
| 26 | 0.13 | 0.04 | CA-CB, CA-CD, CB-CA |
| 27 | 0.13 | 0.01 | CA-CB, CA-CG1, CB-CA, CB-CG1, CB-CG2, CG1-CA, CG1-CB, CG1-CG2, CG2-CA, CG2-CB, CG2-CG1 |
| 28 | 0.15 | 0.02 | CA-CB, CA-CG, CB-CA, CB-CG, CB-CD, CG-CA, CG-CB, CG-CD, CD-CG |
| 29 | 0.15 | 0.09 | CB-CA |
| 30 | - | - | none |
| 31 | 0.16 | 0.02 | CA-CB, CA-CG, CB-CA, CB-CG, CG-CA, CG-CB |
| 32 | 0.13 | 0.05 | CA-CB, CA-CD, CB-CA |
| 33 | 0.14 | 0.03 | CA-CB, CB-CA, CB-CG1, CB-CG2, CG1-CB, CG1-CD1, CG2-CB, CD1-CB, CD1-CG1 |
| 34 | 0.23 | 0.04 | CA-CG, CA-CD, CA-CE, CB-CA, CG-CA, CG-CD, CG-CE, CD-CA, CD-CB, CD-CG, CD-CE, CE-CA, CE-CG, CE-CD |
| 35 | 0.16 | 0.05 | CA-CG, CB-CA, CB-CG, CG-CB |
| 36 | 0.16 | 0.03 | CA-CB, CB-CA, CB-CG2, CG2-CB |
| 37 | 0.21 | 0.04 | CA-C, CA-CB, CB-CA, CB-CG, CG-C, CG-CD |
| **pH 4.5, 25 mM $Ca^{2+}$** | | | |
| 7 | 0.88 | 0.33 | CA-C, CA-CB, CB-CA |
| 8 | - | - | none |
| 9 | 0.64 | 0.44 | CA-CB |
| 10 | 0.31 | 0.08 | CA-CA, CA-C |

| | | | |
|---|---|---|---|
| 11 | 0.19 | 0.03 | CA-CB, CA-CD1, CB-CA, CB-CG1, CB-CD1, CG1-CB, CG1-CG2 |
| 12 | 0.18 | 0.01 | CA-CB, CA-CG1, CA-CG2, CB-CA, CB-CG1, CB-CG2, CG1-CA |
| 13 | 0.18 | 0.01 | CA-CB, CA-CG, CB-CA, CB-CG, CG-CA, CG-CB, |
| 14 | 0.26 | 0.10 | CA-CB, CB-CA |
| 15 | 0.30 | 0.01 | CA-CB, CB-CA |
| 16 | 0.20 | 0.02 | CA-CB, CA-CG1, CA-CG2, CB-CA, CB-CG2, CG1-CA, CG1-CB |
| 17 | 0.18 | 0.01 | CA-CB, CA-CG, CB-CA, CB-CG, CG-CA, CG-CB |
| 18 | - | - | none |
| 19 | 0.18 | 0.01 | CA-CB, CA-CG, CB-CA, CB-CG, CG-CA, CG-CB |
| 20 | 0.18 | 0.01 | CA-CB, CA-CG, CB-CA, CB-CG, CG-CA, CG-CB |
| 21 | 0.18 | 0.01 | CA-CB, CA-CG1, CA-CG2, CB-CA, CB-CG1, CB-CG2, CG1-CA |
| 22 | - | - | none |
| 23 | 0.19 | 0.02 | CA-CB, CB-CA, CB-CG1, CB-CG2, CG1-CA, CG1-CB, CG1-CG2 |
| 24 | 0.17 | 0.04 | CA-CB, CA-CD, CB-CA, CB-CD |
| 25 | 0.17 | 0.04 | CA-CB, CA-CD, CB-CA, CB-CD |
| 26 | 0.17 | 0.04 | CA-CB, CA-CD, CB-CA, CB-CD |
| 27 | 0.18 | 0.01 | CA-CB, CA-CG1, CA-CG2, CB-CA, CB-CG1, CB-CG2, CG1-CA |
| 28 | 0.18 | 0.02 | CA-CB, CA-CG, CB-CA, CB-CG, CG-CA, CG-CB, CG-CD |
| 29 | 0.14 | 0.05 | CA-CB, CB-CA |
| 30 | - | - | none |
| 31 | 0.22 | 0.02 | CA-CB, CA-CG, CB-CA, CB-CG, CG-CA, CG-CB, |
| 32 | 0.17 | 0.04 | CA-CB, CA-CD, CB-CA, CB-CD |
| 33 | 0.19 | 0.02 | CA-CB, CA-CG2, CB-CA, CB-CG1, CB-CG2, CG1-CA, CG1-CB |
| 34 | 0.24 | 0.03 | CA-CB, CA-CG, CA-CD, CA-CE, CB-CA, CB-CD, CG-CA |
| 35 | 0.21 | 0.05 | CA-CB, CA-CG, CB-CG, CG-CB |
| 36 | 0.18 | 0.03 | CA-CB, CB-CA, CB-CG2, CG2-CB |
| 37 | 0.26 | 0.09 | CB-CG, CG-C, CG-CD |

**Table S6**. Water-accessibility values of NL63 ETM at pH 7.4, obtained from water-edited 2D NCA spectra.

| Residue | $S/S_0$ | error |
|---|---|---|
| D7 | - | - |
| D8 | 0.31 | 0.37 |
| N9 | 0.74 | 0.45 |
| G10 | 0.51 | 0.50 |
| I11 | 0.17 | 0.13 |
| V12 | 0.26 | 0.17 |
| L13 | 0.16 | 0.02 |
| N14 | 0.24 | 0.04 |
| S15 | 0.14 | 0.06 |
| I16 | 0.19 | 0.08 |
| L17 | 0.16 | 0.02 |
| W18 | - | - |
| L19 | 0.16 | 0.02 |
| L20 | 0.16 | 0.02 |
| V21 | 0.12 | 0.06 |
| M22 | 0.17 | 0.06 |
| I23 | 0.14 | 0.08 |
| F24 | 0.09 | 0.05 |
| F25 | 0.09 | 0.05 |
| F26 | 0.09 | 0.05 |
| V27 | 0.13 | 0.04 |
| L28 | 0.16 | 0.02 |
| A29 | 0.20 | 0.08 |
| M30 | 0.26 | 0.17 |
| T31 | 0.15 | 0.04 |
| F32 | 0.17 | 0.07 |
| I33 | 0.14 | 0.08 |
| K34 | 0.16 | 0.03 |
| L35 | 0.24 | 0.04 |
| I36 | 0.37 | 0.27 |
| Q37 | 0.29 | 0.07 |

**Table S7.** Detailed parameters for the solid-state NMR experiments.

| Experiment | NMR parameters | Expt. time |
|---|---|---|
| | **NL63 E (1-37), pH 7.4** | |
| 25 ms 2D CC | $B_0$ = 18.8 T, $T_{sample}$=22°C, $\nu_r$ = 10.5 kHz, ns = 32, $t_1$ = 7.5 ms, $\tau_{HC}$ = 0.5 ms, $\tau_{CORD}$ = 25 ms, d1 =1.7 s | 9.5 h |
| 2D NCA | $B_0$ = 18.8 T, $T_{sample}$ = 22°C, $\nu_r$ = 10.5 kHz, ns = 112, $t_1$ = 10 ms, $\tau_{HN}$ = 1 ms, $\tau_{NC}$ = 4.5 ms, $^{13}$C rf carrier = 59 ppm, d1 =2 s | 14 h |
| 3D NCACX | $B_0$ = 18.8 T, $T_{sample}$ =22°C, $\nu_r$ = 14 kHz, ns = 48, SW2 = 7000 Hz, SW1 = 7000 Hz, $t_2$ = 4.5 ms, $t_1$=5.7 ms, $\tau_{HN}$ = 1 ms, $\tau_{NC}$ = 4.5 ms, $\tau_{CORD}$ = 51.4 ms, $^{13}$C rf carrier = 59 ppm, d1 =1.7 s | 122 h |
| 3D NCOCX | $B_0$ = 18.8 T, $T_{sample}$ =22°C, $\nu_r$ = 14 kHz, ns = 128, SW2 = 3500 Hz, SW1 = 3500 Hz, $t_2$ = 5.1 ms, $t_1$= 6 ms, $\tau_{HN}$ = 1 ms, $\tau_{NC}$ = 4.5 ms, $\tau_{CORD}$ = 51.4 ms, $^{13}$C rf carrier = 175 ppm, d1 =1.7 s | 96 h |
| 3D CONCA | $B_0$ = 18.8 T, $T_{sample}$ =22°C, $\nu_r$ = 14 kHz, ns = 24, SW2 = 7000 Hz, SW1 = 4666.7 Hz, $t_2$ = 6.4 ms, $t_1$=6.4 ms, $\tau_{HC}$ = 0.6 ms, $\tau_{CoN}$ = 4.5 ms, $^{13}$C rf carrier = 175 ppm (+ freq. jump to 59 ppm), d1 =1.85 s | 68 h |
| | **NL63 ETM (7-37), pH 7.4** | |
| 24 ms 2D CC | $B_0$ = 18.8 T, $T_{sample}$ =25°C, $\nu_r$ = 14 kHz, ns = 96, $t_1$ = 5 ms, $\tau_{HC}$ = 0.5 ms, $\tau_{CORD}$ = 24 ms, d1 =1.7 s | 19 h |
| 2D NCA | $B_0$ = 18.8 T, $T_{sample}$ =25°C, $\nu_r$ = 14 kHz, ns = 192, $t_1$ = 9 ms, $\tau_{HN}$ = 1 ms, $\tau_{NC}$ = 4.5 ms, $^{13}$C rf carrier = 59 ppm, d1 =2 s | 14 h |
| 3D NCACX | $B_0$ = 18.8 T, $T_{sample}$ =25°C, $\nu_r$ = 14 kHz, ns = 64, SW2=7000 Hz, SW1 = 3500 Hz, $t_2$ = 4.5 ms, $t_1$=7.1 ms, $\tau_{HN}$ = 1 ms, $\tau_{NC}$ = 4.5 ms, $\tau_{CORD}$ = 51.4 ms, $^{13}$C rf carrier= 59 ppm, d1 =1.7 s | 130 h |
| 3D NCOCX | $B_0$ = 18.8 T, $T_{sample}$ =25°C, $\nu_r$ = 14 kHz, ns = 160, SW2 = 3500 Hz, SW1 = 3500Hz, $t_2$ = 5.1 ms, $t_1$=6 ms, $\tau_{HN}$ = 1 ms, τNC = 4.5 ms, $\tau_{CORD}$ = 62 ms, $^{13}$C rf carrier = 175 ppm, d1 =1.7 s | 121 h |
| 3D CONCA | $B_0$ = 18.8 T, $T_{sample}$ =25°C, $\nu_r$ = 14 kHz, ns = 24, SW2 = 7000 Hz, SW1 = 4666.7 Hz, $t_2$ = 6.4 ms, $t_1$=6.4 ms, $\tau_{HC}$ = 0.6 ms, $\tau_{CoN}$ = 4.5 ms, $^{13}$C rf carrier = 175 ppm (+ freq. jump to 59 ppm), d1 =1.8 s | 66 h |
| Water-edited NCA | $B_0$ = 18.8 T, $T_{sample}$ =25°C, $\nu_r$ = 14 kHz, $ns_{total}$ = 704, $\tau_{1H\text{-}SD}$ = 9 ms, $^1$H T2 filter = 2.14 ms, $p_{soft\text{-}pulse}$= 2 ms, 1H rf carrier = 4.74 ppm, $t_1$ = 9 ms, $\tau_{HN}$ = 1 ms, $\tau_{NC}$ = 4.5 ms, $^{13}$C rf carrier = 59 ppm, d1 =2 s | 2 x 26 h |
| Water-edited 2D CC | $B_0$ = 18.8 T, $T_{sample}$ =25°C, $\nu_r$ = 14 kHz, $ns_{total}$ = 280, $\tau_{1H\text{-}SD}$ = 9 ms, $^1$H T2 filter = 2.71 ms, $p_{soft\text{-}pulse}$= 2.57 ms, $^1$H rf carrier = 4.74 ppm, $t_1$ = 5 ms, $\tau_{HC}$ = 0.5 ms, $\tau_{CORD}$ = 24 ms, d1 =1.7 s | 55 h |
| Water-edited 1D $^{13}$C spectra | $B_0$ = 18.8 T, $T_{sample}$ =25°C, $\nu_r$ = 14 kHz, ns = 1024, $\tau_{1H\text{-}SD}$ = 0, 9 and 100 ms, $^1$H T2 filter = 2.71 ms, $p_{soft\text{-}pulse}$= 2.57 ms, $^1$H rf carrier = 4.74 ppm, $\tau_{HC}$ = 0.5 ms, d1 =1.7 s | 0.5 h each |
| Lipid-edited 1D $^{13}$C spectra | $B_0$ = 18.8 T, $T_{sample}$ =25°C, $\nu_r$ = 14 kHz, ns = 8192, $\tau_{1H\text{-}SD}$ = 0, 4, 9, 16, 49 and 100 ms, $^1$H T2 filter = 3.14 ms, $p_{soft\text{-}pulse}$ = 3 ms, $^1$H rf carrier = 1.3 ppm, $\tau_{HC}$ = 0.5 ms, d1 =1.8 s | 4.5 h each |
| | **NL63 ETM (7-37), pH 4.5** | |

| | | |
|---|---|---|
| 24 ms 2D CC | $B_0$ = 18.8 T, $T_{sample}$ =*25*°C, $\nu_r$ = 14 kHz, ns = 96, $t_1$ = 6 ms, $\tau_{HC}$ = 0.5 ms, $\tau_{CORD}$ = 24 ms, d1 =1.7 s | 23 h |
| 2D NCA | $B_0$ = 18.8 T, $T_{sample}$ =25°C, $\nu_r$ = 14 kHz, ns = 192, $t_1$ = 9 ms, $\tau_{HN}$ = 1 ms, $\tau_{NC}$ = 4.5 ms, $^{13}C$ rf carrier = 59 ppm, d1 =2 s | 14 h |
| Water-edited 2D CC | $B_0$ = 18.8 T, $T_{sample}$ =25°C, $\nu_r$ = 14 kHz, $ns_{total}$ = 248, $\tau_{1H\text{-}SD}$ = 9 ms, $^1H$ T2 filter = 2.71 ms, $p_{soft\text{-}pulse}$= 2.57 ms, $^1H$ rf carrier = 4.70 ppm, $t_1$ = 6 ms, $\tau_{HC}$ = 0.5 ms, $\tau_{CORD}$ = 24 ms, d1 =1.75 s | 61 h |
| Water-edited 1D $^{13}C$ spectra | $B_0$ = 18.8 T, $T_{sample}$ =25°C, $\nu_r$ = 14 kHz, ns = 1024, $\tau_{1H\text{-}SD}$ = 0, 4, 9, 16, 49 and 100 ms, $^1H$ T2 filter = 2.71 ms, $^1H$ $p_{soft\text{-}pulse}$= 2.57 ms, $^1H$ rf carrier = 4.70 ppm, $\tau_{HC}$ = 0.5 ms, d1 =1.8 s | 0.5 h each |
| Lipid-edited 1D $^{13}C$ spectra | $B_0$ = 18.8 T, $T_{sample}$ =25°C, $\nu_r$ = 14 kHz, ns = 8192, $\tau_{1H\text{-}SD}$ = 0, 4, 9, 16, 49 and 100 ms, $^1H$ $T_2$ filter = 3.14 ms, $^1H$ $p_{soft\text{-}pulse}$= 3 ms, $^1H$ rf carrier = 1.27 ppm, $\tau_{HC}$ = 0.5 ms, d1 =1.8 s | 4.5 h each |

Definitions of symbols are: $B_0$ = magnetic field; $T_{sample}$ = sample temperature calculated from the chemical shift of water $^1H$ signal; $\nu_r$ = MAS; ns = number of scans per free induction decay; $\tau_{1H\text{-}SD}$ = $^1H$ spin diffusion mixing time; $p_{soft\text{-}pulse}$ = $^1H$ shaped pulse length for selective excitation of water or lipids; max $t_1$ = maximum $t_1$ evolution time; max $t_2$: maximum $t_2$ evolution time; $\tau_{HC}$= $^1H$-$^{13}C$ CP contact time; $\tau_{HN}$= $^1H$-$^{15}N$ CP contact time; $\tau_{NC}$= $^{15}N$-$^{13}C$ CP contact time; $\tau_{CoN}$= CO-$^{15}N$ CP contact time; $\tau_{CORD}$ = $^{13}C$ CORD mixing time; d1= recycle delay.
